# Comparative Analysis of Resmetirom vs. FGF21 Analogs vs. GLP-1 Agonists in MASLD and MASH: Network Meta-Analysis of Clinical Trials

**DOI:** 10.3390/biomedicines12102328

**Published:** 2024-10-14

**Authors:** Hazem Ayesh, Azizullah Beran, Sajida Suhail, Suhail Ayesh, Kevin Niswender

**Affiliations:** 1 Deaconess Health System, Evansville, IN 47708, USA; 2 Division of Gastroenterology and Hepatology, Indiana University School of Medicine, Indianapolis, IN 46202, USA; 3 Gene Medical Labs, Gaza 00972, Palestine; 4 Department of Medicine, Division of Diabetes, Endocrinology, and Metabolism, Vanderbilt University Medical Center, Nashville, TN 37232, USA

**Keywords:** Resmetirom, MASLD, MASH, FGF21, GLP-1

## Abstract

Introduction: Metabolic Dysfunction-Associated Steatotic Liver Disease (MASLD) and Metabolic-Dysfunction Associated Steatohepatitis (MASH) are linked to obesity, type 2 diabetes, and metabolic syndrome, increasing liver-related morbidity and cardiovascular risk. Recent therapies, including Resmetirom, FGF21 analogs, and GLP-1 agonists, have shown promise. This network meta-analysis evaluates their comparative efficacy and safety. Methods: A literature search was conducted across PubMed, Scopus, Web of Science, and Cochrane Library. Included clinical trials addressed MASLD or MASH with Resmetirom, FGF21 analogs, or GLP-1 agonists. Statistical analyses used a random-effects model, calculating mean differences (MD) and relative risks (RR), with heterogeneity assessed using τ^2^, I^2^, and Q statistics. Results: MASH resolution was significantly higher for FGF21 (RR 4.84, 95% CI: 2.59 to 9.03), Resmetirom showed the most significant reduction in MRI-PDFF (MD −18.41, 95% CI: −23.60 to −13.22) and >30% fat reduction (RR 3.56, 95% CI: 2.41 to 5.26). Resmetirom significantly reduced ALT (MD −15.71, 95% CI: −23.30 to −8.13), AST (MD −12.28, 95% CI: −21.07 to −3.49), and GGT (MD −19.56, 95% CI: −34.68 to −4.44). FGF21 and GLP-1 also reduced these markers. Adverse events were significantly higher with Resmetirom (RR 1.47, 95% CI: 1.24 to 1.74), while GLP-1 and FGF21 showed non-significant trends towards increased risk. Conclusions: Resmetirom and FGF21 show promise in treating MASLD and MASH, with Resmetirom particularly effective in reducing liver fat and improving liver enzymes. GLP-1 agonists also show benefits but to a lesser extent. Further long-term studies are needed to validate these findings and assess cost-effectiveness.

## 1. Introduction

Metabolic Dysfunction-Associated Steatotic Liver Disease (MASLD) and Metabolic Dysfunction-Associated Steatohepatitis (MASH) are increasingly recognized as significant global health concerns, affecting millions of individuals worldwide [1]. MASLD, which encompasses a spectrum of liver conditions ranging from simple steatosis to MASH, is closely associated with obesity, type 2 diabetes, and metabolic syndrome. These conditions not only increase the risk of liver-related morbidity and mortality but are also associated with cardiovascular diseases and other metabolic complications, significantly burdening healthcare systems [2]. The economic impact is substantial, with direct medical costs attributed to MASLD and MASH estimated to be in the billions of dollars annually in the United States alone. Moreover, patients with MASLD and MASH often face higher healthcare utilization, including increased hospitalizations and outpatient visits, further straining healthcare resources [3].

**Figure 1:**
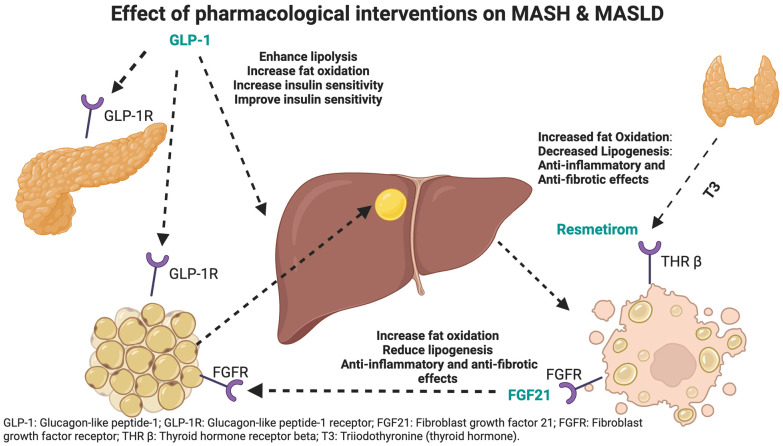
Mechanisms of action for pharmacological interventions in MASH and MASLD. Created in BioRender. Ayesh, H. (2024) BioRender.com/f74r889, accessed on 5 October 2024.

Recent advancements in the pharmacological treatment of MASLD and MASH have shown promising results, particularly with medications such as Resmetirom and Fibroblast Growth Factor 21 (FGF21) analogs. Resmetirom, a selective thyroid hormone receptor-β agonist, enhances hepatic fat metabolism and significantly reduces liver lipid content, making it a potent agent for liver fat reduction and improved liver function [4]. On the other hand, FGF21 analogs, such as Pegbelfermin, Efruxifermin, and Pegozafermin, have demonstrated strong anti-inflammatory and insulin-sensitizing effects in the liver [5]. FGF21 enhances fatty acid oxidation, reduces hepatic lipid accumulation, and improves insulin sensitivity by activating the FGFR1c and β-Klotho (KLB) receptor complex. This activation increases energy expenditure and reduces lipotoxicity, alleviating hepatic steatosis and improving liver function [5]. Comparatively, Glucagon-like Peptide-1 (GLP-1) agonists, such as liraglutide and semaglutide, primarily work by enhancing insulin secretion, inhibiting glucagon release, promoting weight loss, and improving lipid levels. These mechanisms are beneficial for reducing liver fat and improving overall metabolic health [6]. The distinct mechanisms and therapeutic effects of these agents highlight the evolving landscape of treatment options for liver diseases. Distinctive mechanisms of action are depicted in Figure 1.

Conducting a meta-analysis on the efficacy and safety of novel therapeutic agents like Resmetirom, FGF21 analogs, and GLP-1 agonists for MASLD and MASH is essential for offering a comparative evaluation among these therapeutic options. Despite numerous clinical trials, comprehensive comparative analyses that offer clear guidance for clinical practice are limited. Our meta-analysis aims to address this gap by systematically evaluating available evidence on these therapies, providing insights into their relative benefits and risks. This analysis will aid clinicians in making informed decisions tailored to individual patient profiles, ultimately improving the management of MASLD and MASH.

## 2. Methods

The study protocol is registered on the Open Science Framework (OSF) at (https://osf.io/hxvp3, accessed on 15 June 2024) [7]. The results of this meta-analysis adhere to the Preferred Reporting Items for Systematic Reviews and Meta-Analyses (PRISMA) guidelines [8]. The PRISMA checklist was followed throughout the study.

### 2.1. Search Strategy

A comprehensive literature search was conducted across PubMed, Scopus, Web of Science, and the Cochrane Library to identify relevant studies from inception to 8 June 2024. The search terms included: (“Resmetirom” OR “MGL-3196” OR “Thyroid hormone receptor-β agonist”) OR (“FGF21 analogs” OR “Pegozafermin” OR “BIO89-100” OR “Pegbelfermin” OR “BMS-986036” OR “Efruxifermin” OR “AKR-001”) OR (“GLP-1 agonists” OR “liraglutide” OR “semaglutide” OR “dulaglutide”) AND (“MASLD” OR “MASH” OR “NASH” OR “MAFLD” OR “NAFLD”) AND (“clinical trials” OR “randomized controlled trials” OR “RCTs” OR “trial”). The detailed search strategy is reported in Supplement S1.

### 2.2. Screening Process

Two authors (HA and SS) independently screened the titles and abstracts of retrieved studies. Full-text articles were reviewed for eligibility. Discrepancies were resolved through discussion or by consulting a third author (SA) if necessary.

### 2.3. Study Selection

We included clinical trials addressing MASLD or MASH investigating Resmetirom, FGF21 analogs (Pegozafermin, Pegbelfermin, Efruxifermin), GLP-1 agonists, or combinations thereof, reporting outcomes related to liver histology, liver fat reduction, and biochemical markers. Studies were excluded if they focused on pediatric populations, did not report relevant outcomes, or were non-comparative studies or conference abstracts without full-text availability. Post-hoc analysis and extension studies were excluded.

### 2.4. Data Extraction

Data extracted from each study included participant demographics, baseline characteristics, intervention details, and outcome measures. Specifically, data on age, sex, body mass index (BMI), diabetes duration, liver enzyme levels, and liver fat content were collected. Information on study design, duration, and adverse events was also extracted. For studies reporting medians and interquartile ranges, these were converted to means and standard deviations using the method by Wan et al. [9]. Combined means and standard deviations were calculated following the Cochrane Handbook for Systematic Reviews of Interventions guidelines [10].

### 2.5. Statistical Analysis

We implemented a network meta-analysis to compare multiple treatments by analyzing data from various studies, allowing for both direct and indirect comparisons. This method synthesizes evidence to determine the relative effectiveness of each treatment, even if some treatments were not directly compared in any individual study [11]. Statistical analyses were conducted using a random-effects model to account for heterogeneity among studies. Mean differences (MD) for continuous outcomes and relative risks (RR) for dichotomous outcomes were calculated. We used standardized mean difference (SMD) for outcomes with different measurement methods. Heterogeneity was evaluated using τ^2^ (tau-squared), I^2^ statistics, and Q statistics [12]. All analyses were performed using RStudio with the meta and netmeta packages [13,14]. P-scores were used to rank treatments based on their estimated effect sizes, with higher P-scores associated with better outcomes [15]. The outcomes evaluated include the following: MASH resolution is defined as the complete absence of ballooning and mild or no inflammation without worsening fibrosis. Improvement in fibrosis refers to a reduction of at least one stage in fibrosis severity without worsening MASH. MRI-PDFF (Magnetic Resonance Imaging-Proton Density Fat Fraction) measures the alteration in liver fat content over the study period, with a specific evaluation for more than 30% fat reduction. VCTE (Vibration-Controlled Transient Elastography) assesses changes in liver stiffness, a surrogate marker for fibrosis. Changes in liver enzyme levels include ALT (Alanine Aminotransferase), AST (Aspartate Aminotransferase), and GGT (Gamma-Glutamyl Transferase), which are indicators of liver injury. Safety outcomes include adverse events, treatment discontinuation due to TEAEs (treatment-emergent adverse events), and the incidence of nausea and vomiting among participants. Sensitivity analysis and meta-regression were performed using different statistical techniques.

### 2.6. Bias Assessment and Certainty of Evidence

The risk of bias was assessed using the RoB 2 (Risk of Bias 2) tool, which evaluates bias across five domains: randomization, deviations from intended interventions, missing outcome data, outcome measurement, and selection of reported results [16]. Two authors (HA and SS) independently assessed the risk of bias. Disagreements were resolved through discussion or by consulting a third author (SA). The certainty of evidence was assessed using the Confidence in Network Meta-Analysis (CINeMA) framework [17].

## 3. Results

### 3.1. Study Characteristics

**Figure 2:**
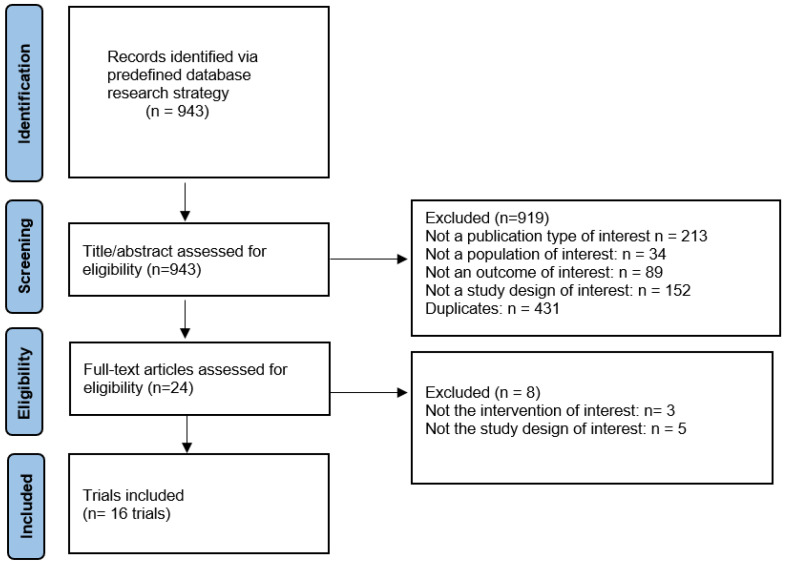
PRISMA flowchart for study selection.

**Figure 3:**
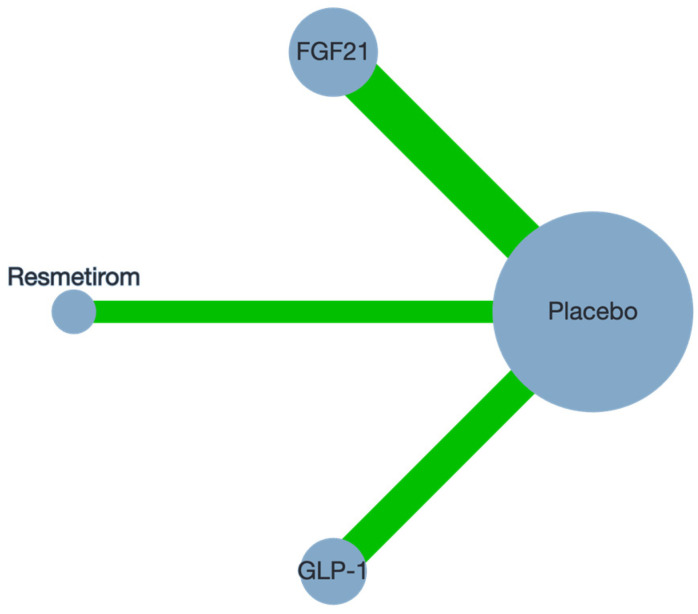
Network plot of treatment comparisons for NASH resolution. This network plot shows the direct comparisons among Resmetirom, FGF21, GLP-1, and placebo in studies for NASH resolution. Node size reflects the number of studies involving each treatment, while edge thickness indicates the number of direct comparisons between treatments.

A total of 16 studies (Table 1) with a total of 3535 participants were included in the analysis [4,18,19,20,21,22,23,24,25,26,27,28,29,30,31,32] (Figure 2) (Supplement S1). The network for the included interventions is presented in Figure 3. The mean age of participants was 55.60 years (SD 11.35), with a mean BMI of 35.62 kg/m^2^ (SD 6.43). Liver function tests showed a mean ALT level of 42.76 U/L (SD 44.18) and a mean AST level of 33.09 U/L (SD 37.28). Glycemic control, measured by HbA1c, had a mean of 6.55% (SD 1.12), with 48.6% of participants being male (Supplement S2 and S3); baseline characteristics are summarized in Table 2. The risk of bias was generally low, with some domains having some concerns (Supplement S4). Publication bias was generally low except for VCTE, which showed a high risk of bias (Supplement S6). The certainty of evidence was generally low due to concerns of incoherence and imprecision (Supplement S8).

### 3.2. Sensitivity Analysis and Meta-Regression

To ensure the robustness of our findings and validate the transitivity assumption, we conducted a meta-regression analysis. Sensitivity analysis was further performed using a Bayesian approach to strengthen the reliability of our results. In our meta-regression, we employed the Bayesian method, incorporating key variables such as HbA1c, age, and gender (male) for a comprehensive subgroup analysis. The Bayesian analysis revealed consistent treatment rankings across all outcomes, underscoring the reliability and stability of the results. Additionally, the subgroup analysis confirmed the uniformity of treatment rankings across different strata, as detailed below (Supplement S10).

### 3.3. Biopsy Outcomes

#### 3.3.1. NASH Resolution

**Figure 4:**
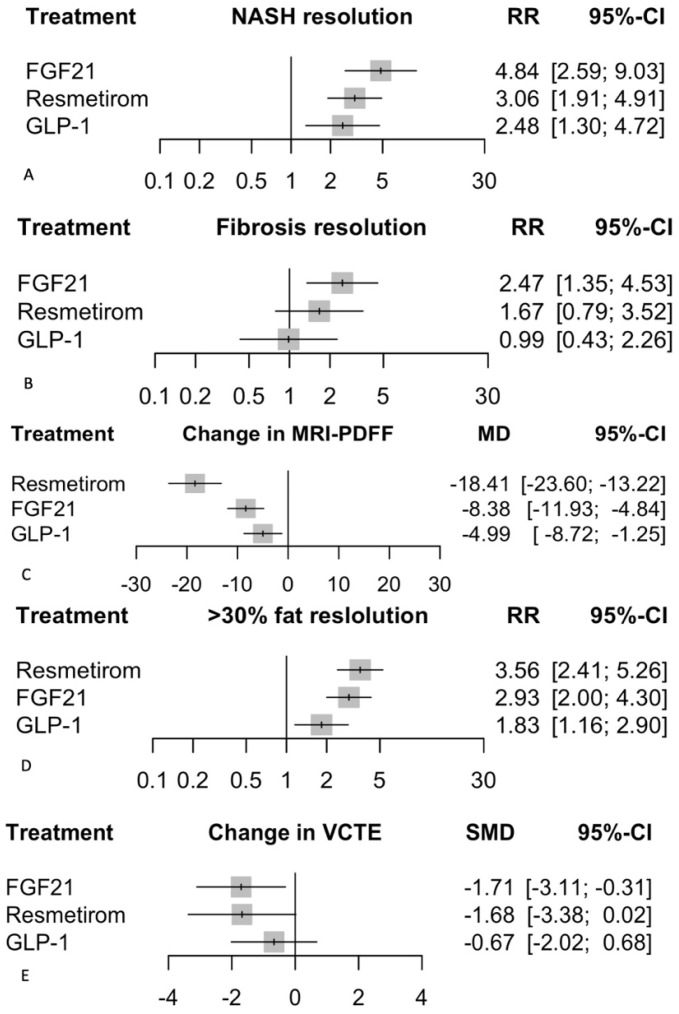
Results of biopsy and imaging outcomes. This figure presents a series of forest plots comparing the efficacy of FGF21, Resmetirom, and GLP-1 treatments against placebo in patients with MASH/MASLD. The outcomes measured are: (**A**) NASH resolution: The relative risk (RR) with 95% confidence intervals (CI) shows the likelihood of NASH resolution for each treatment compared to placebo. (**B**) Improvement in fibrosis: The RR with 95% CI indicates the likelihood of fibrosis resolution for each treatment compared to placebo. (**C**) Change in MRI-PDFF: The mean difference (MD) with 95% CI represents the change in MRI-estimated proton density fat fraction (MRI-PDFF) for each treatment compared to placebo. (**D**) >30% fat resolution: The RR with 95% CI shows the likelihood of achieving more than 30% fat resolution for each treatment compared to placebo. (**E**) Change in VCTE: The standardized mean difference (SMD) with 95% CI indicates the change in vibration-controlled transient elastography (VCTE) scores for each treatment compared to placebo. Grey squares represent effect estimates; horizontal lines show 95% CIs.

In the random effects model, the relative risk (RR) of NASH resolution compared to placebo was significantly higher for all treatments (Figure 4): FGF21 (RR 4.84, 95% CI: 2.59 to 9.03, *p* < 0.0001), GLP-1 agonists (RR 2.48, 95% CI: 1.30 to 4.72, *p* = 0.006), and Resmetirom (RR 3.06, 95% CI: 1.91 to 4.91, *p* < 0.0001). The heterogeneity analysis showed an I^2^ of 11.70%, indicating low to moderate heterogeneity. The tests for heterogeneity within designs (Q = 6.79, df = 6, *p* = 0.34) and between designs (Q = 0.00, df = 0) were not significant, suggesting consistent treatment effects across studies. P-scores for ranking the treatments were as follows: FGF21 (0.93), Resmetirom (0.61), and GLP-1 (0.46), indicating that FGF21 agonists had the highest probability of being the most effective treatment for NASH resolution. There was no difference in the subgroups (Supplement S10) in the Bayesian analysis, indicating the robustness of the results.

#### 3.3.2. Improvement in Fibrosis

In the random effects model assessing the improvement in fibrosis, FGF21 demonstrated a significant relative risk (RR) of 2.47 (95% CI: 1.35 to 4.53, *p* = 0.003), which was statistically significant in comparison to placebo (Figure 4). GLP-1, however, showed no significant effect with an RR of 0.99 (95% CI: 0.43 to 2.26, *p* = 0.97), and Resmetirom had an RR of 1.67 (95% CI: 0.79 to 3.52, *p* = 0.18), also not reaching significance. The heterogeneity analysis revealed moderate to substantial heterogeneity with I^2^ of 62.50%. The tests for heterogeneity within designs were significant (Q = 18.65, df = 7, *p* = 0.009), suggesting variability in the effect estimates. P-scores, which rank treatments based on their effectiveness, were highest for FGF21 (0.92), followed by Resmetirom (0.65) and GLP-1 (0.23), indicating FGF21 analogs as the most effective treatment for fibrosis improvement among the interventions studied. There was no difference in the subgroups (Supplement S10) in the Bayesian analysis, indicating the robustness of the results.

### 3.4. Imaging Outcomes

#### 3.4.1. Change in MRI-PDFF

In the random effects model assessing the change in MRI-PDFF, Resmetirom demonstrated the most significant effect with a mean difference (MD) of −18.41 (95% CI: −23.60 to −13.22, *p* < 0.0001) compared to placebo, indicating a substantial reduction in liver fat content (Figure 4). FGF21 also showed a significant effect with an MD of −8.38 (95% CI: −11.93 to −4.84, *p* < 0.0001), followed by GLP-1 with an MD of −4.99 (95% CI: −8.72 to −1.25, *p* = 0.009). The heterogeneity analysis revealed significant variability across studies, with an I^2^ of 89.00%. Tests of heterogeneity within designs were significant (Q = 72.44, df = 8, *p* < 0.0001), indicating considerable inconsistency among the study results. The P-scores, which rank treatments based on their effectiveness, were highest for Resmetirom (1.00), followed by FGF21 (0.63) and GLP-1 (0.36). There was no difference in the subgroups (Supplement S10) in the Bayesian analysis, indicating the robustness of the results.

#### 3.4.2. >30% Fat Reduction on MRI-PDFF

In the random effects model assessing a >30% reduction in fat on MRI-PDFF, Resmetirom showed the most significant effect with a relative risk (RR) of 3.56 (95% CI: 2.41 to 5.26, *p* < 0.0001) compared to placebo (Figure 4). FGF21 also demonstrated a significant effect with an RR of 2.93 (95% CI: 2.00 to 4.30, *p* < 0.0001), followed by GLP-1 with an RR of 1.83 (95% CI: 1.16 to 2.90, *p* = 0.010). The heterogeneity analysis revealed moderate heterogeneity, with an I^2^ of 15.20%. The tests for heterogeneity within designs (Q = 10.62, df = 9, *p* = 0.30) were not significant, suggesting consistency among the study results. The P-scores, which rank treatments based on their effectiveness, were highest for Resmetirom (0.91), followed by FGF21 (0.73) and GLP-1 (0.36). There was no difference in the subgroups (Supplement S10) in the Bayesian analysis, indicating the robustness of the results.

#### 3.4.3. Change in VCTE

In the random effects model assessing change in VCTE, FGF21 demonstrated a significant standardized mean difference (SMD) of −1.71 (95% CI: −3.11 to −0.31, *p* = 0.017) compared to placebo, while Resmetirom showed a borderline significant effect with an SMD of −1.68 (95% CI: −3.38 to 0.02, *p* = 0.053) (Figure 4). GLP-1 agonists did not show a significant effect, with an SMD of −0.67 (95% CI: −2.02 to 0.68, *p* = 0.33). The heterogeneity analysis indicated substantial variability with an I^2^ of 73.10%. Tests of heterogeneity within designs were significant (Q = 26.03, df = 7, *p* = 0.0005), indicating inconsistency among the study results. P-scores ranked FGF21 (0.78) and Resmetirom (0.76) as the most effective treatments for reducing liver stiffness measured by VCTE, followed by GLP-1 (0.39).

### 3.5. Biochemical Markers

#### 3.5.1. Change in ALT

**Figure 5:**
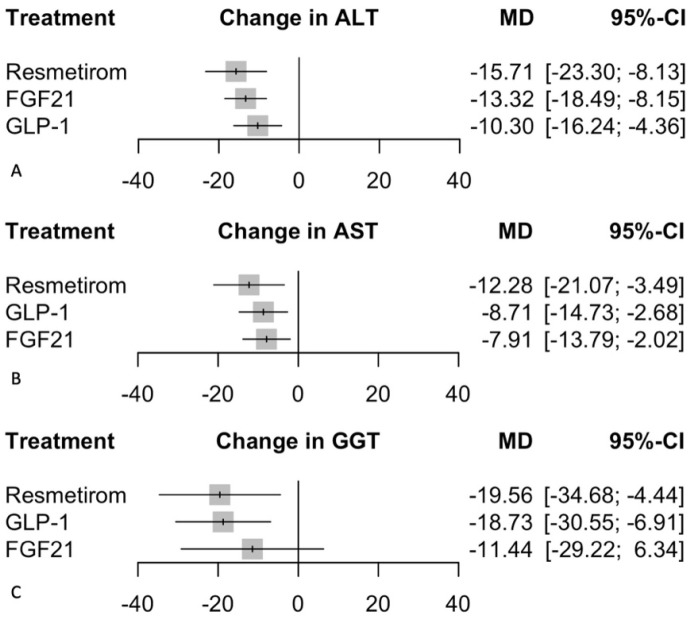
Changes in liver enzymes. This figure presents a series of forest plots comparing the efficacy of FGF21, Resmetirom, and GLP-1 treatments against placebo in patients with MASH/MASLD. The outcomes measured are (**A**) Change in ALT: The mean difference (MD) with 95% confidence intervals (CI) shows the change in alanine aminotransferase (ALT) levels for each treatment compared to placebo. (**B**) Change in AST: The MD with 95% CI indicates the change in aspartate aminotransferase (AST) levels for each treatment compared to placebo. (**C**) Change in GGT: The MD with 95% CI represents the change in gamma-glutamyl transferase (GGT) levels for each treatment compared to placebo. Grey squares represent effect estimates; horizontal lines show 95% CIs.

In the random effects model assessing the change in ALT, Resmetirom demonstrated the most significant effect with a mean difference (MD) of −15.71 (95% CI: −23.30 to −8.13, *p* < 0.0001) compared to placebo (Figure 5). FGF21 also showed a significant reduction in ALT with an MD of −13.32 (95% CI: −18.49 to −8.15, *p* < 0.0001), followed by GLP-1 with an MD of −10.30 (95% CI: −16.24 to −4.36, *p* = 0.0007). The heterogeneity analysis revealed moderate heterogeneity with an I^2^ of 55.80%. Tests of heterogeneity within designs were significant (Q = 22.61, df = 10, *p* = 0.012), indicating variability among the study results. The P-scores, which rank treatments based on their effectiveness, were highest for Resmetirom (0.85), followed by FGF21 (0.69) and GLP-1 (0.45). There was no difference in the subgroups (Supplement S10) in the Bayesian analysis, indicating the robustness of the results.

#### 3.5.2. Change in AST

In the random effects model assessing the change in AST, Resmetirom demonstrated the most significant reduction with a mean difference (MD) of −12.28 (95% CI: −21.07 to −3.49, *p* = 0.006) compared to placebo (Figure 5). GLP-1 also showed a significant reduction with an MD of −8.71 (95% CI: −14.73 to −2.68, *p* = 0.005), followed by FGF21 with an MD of −7.91 (95% CI: −13.79 to −2.02, *p* = 0.009). The heterogeneity analysis revealed substantial variability across studies, with an I^2^ of 77.80%. Tests of heterogeneity within designs were significant (Q = 45.07, df = 10, *p* < 0.0001), indicating inconsistency among the study results. The P-scores, which rank treatments based on their effectiveness, were highest for Resmetirom (0.84), followed by GLP-1 (0.61) and FGF21 (0.54). There was no difference in the subgroups (Supplement S10) in the Bayesian analysis, indicating the robustness of the results.

#### 3.5.3. Change in GGT

In the random effects model assessing the change in GGT, Resmetirom demonstrated a significant reduction with a mean difference (MD) of −19.56 (95% CI: −34.68 to −4.44, *p* = 0.011) compared to placebo (Figure 5). GLP-1 also showed a significant reduction with an MD of −18.73 (95% CI: −30.55 to −6.91, *p* = 0.002), while FGF21 did not show a significant effect with an MD of −11.44 (95% CI: −29.22 to 6.34, *p* = 0.207). The heterogeneity analysis revealed substantial variability across studies, with an I^2^ of 75.50%. Tests of heterogeneity within designs were significant (Q = 20.41, df = 5, *p* = 0.001), indicating inconsistency among the study results. The P-scores, which rank treatments based on their effectiveness, were highest for Resmetirom (0.76), followed closely by GLP-1 (0.74), then FGF21 (0.47). There was no difference in the subgroups (Supplement S10) in the Bayesian analysis, indicating the robustness of the results.

### 3.6. Safety Outcomes

#### 3.6.1. Adverse Events

**Figure 6:**
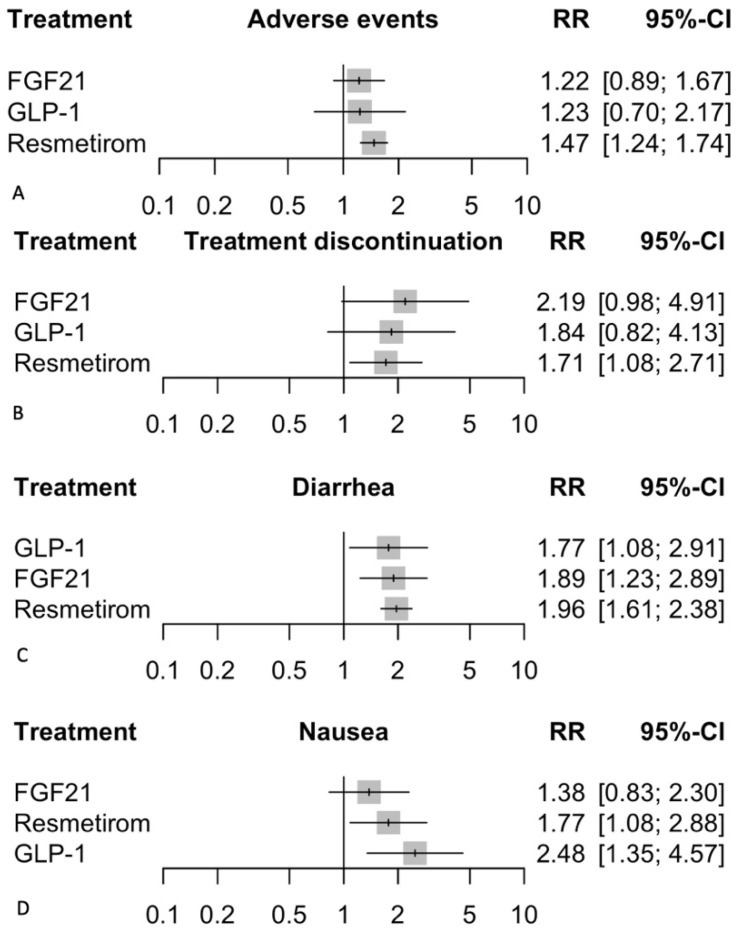
Safety outcomes. This figure presents a series of forest plots comparing the safety of FGF21, GLP-1, and Resmetirom treatments against placebo in MASH/MASLD. The outcomes measured are (**A**) Adverse events: The relative risk (RR) with 95% confidence intervals (CI) shows the likelihood of experiencing adverse events for each treatment compared to placebo. (**B**) Treatment discontinuation: The RR with 95% CI indicates the likelihood of treatment discontinuation for each treatment compared to placebo. (**C**) Diarrhea: The RR with 95% CI represents the likelihood of experiencing diarrhea for each treatment compared to placebo. (**D**) Nausea: The RR with 95% CI shows the likelihood of experiencing nausea for each treatment compared to placebo. Grey squares represent effect estimates; horizontal lines show 95% CIs.

In the random effects model assessing adverse events, Resmetirom showed a significant increase in the risk of adverse events with a relative risk (RR) of 1.47 (95% CI: 1.24 to 1.74, *p* < 0.0001) compared to placebo (Figure 6). GLP-1 agonists did not show a significant effect, with an RR of 1.23 (95% CI: 0.70 to 2.17, *p* = 0.474), indicating a non-significant trend towards increased risk. FGF21 also showed a non-significant increase in the risk of adverse events with an RR of 1.22 (95% CI: 0.89 to 1.67, *p* = 0.220). The heterogeneity analysis revealed no significant heterogeneity, with I^2^ = 0%. Tests for heterogeneity within designs were not significant (Q = 2.29, df = 10, *p* = 0.994), indicating consistency among the study results. The P-scores, which rank treatments based on their effectiveness with higher scores indicating more favorable outcomes, were highest for FGF21 (0.4892), followed by GLP-1 (0.4821), and Resmetirom (0.1445). There was no difference in the subgroups (Supplement S10), indicating the robustness of the results.

#### 3.6.2. Treatment Discontinuation

In the random effects model assessing treatment discontinuation, Resmetirom had a significant increase in the risk of discontinuation with a relative risk (RR) of 1.71 (95% CI: 1.08 to 2.71, *p* = 0.022) compared to placebo (Figure 6). GLP-1 agonists did not show a significant effect with an RR of 1.84 (95% CI: 0.82 to 4.13, *p* = 0.142), although there was a trend towards increased risk. FGF21 showed a non-significant increase in the risk of discontinuation with an RR of 2.19 (95% CI: 0.98 to 4.91, *p* = 0.058). The heterogeneity analysis revealed no significant heterogeneity, with I^2^ = 0%. Tests for heterogeneity within designs were not significant (Q = 3.20, df = 10, *p* = 0.976), indicating consistency among the study results. The P-scores, which rank treatments based on their effectiveness with higher scores indicating more favorable outcomes, were highest for Resmetirom (0.42), followed by GLP-1 (0.38), and FGF21 (0.24). There was no difference in the subgroups (Supplement S10) in the Bayesian analysis, indicating the robustness of the results.

#### 3.6.3. Nausea

In the random effects model assessing adverse events, GLP-1 agonists showed the highest significant increase in risk with a relative risk (RR) of 2.48 (95% CI: 1.35 to 4.57, *p* = 0.0035) compared to placebo (Figure 6). Resmetirom followed with a significant increase in risk, presenting an RR of 1.77 (95% CI: 1.08 to 2.88, *p* = 0.0223). FGF21 exhibited a non-significant increase in the risk of nausea with an RR of 1.38 (95% CI: 0.83 to 2.30, *p* = 0.2130). The heterogeneity analysis indicated moderate heterogeneity with tau^2 = 0.0957 and I^2 = 39.4%. Tests for heterogeneity within designs were not significant (Q = 14.86, df = 9, *p* = 0.0949), suggesting consistency among the study results. The P-scores, which rank treatments based on their likelihood of causing adverse events, were highest for FGF21 (0.5955), followed by Resmetirom (0.3532) and GLP-1 (0.0911). This ranking indicates that GLP-1 had the highest risk of nausea, followed by Resmetirom, with FGF21 showing the least increase in risk compared to placebo. There was no difference in the subgroups (Supplement S10) in the Bayesian analysis, indicating the robustness of the results. However, it showed a significantly higher incidence in those over 55 years old across all treatment groups.

#### 3.6.4. Diarrhea

In the random effects model assessing the incidence of diarrhea, Resmetirom demonstrated a significant increase in the risk with a relative risk (RR) of 1.96 (95% CI: 1.61 to 2.38, *p* < 0.0001) compared to placebo (Figure 6). FGF21 also showed a significant increase in the risk of diarrhea with an RR of 1.89 (95% CI: 1.23 to 2.89, *p* = 0.004), followed by GLP-1 with an RR of 1.77 (95% CI: 1.08 to 2.91, *p* = 0.024). The heterogeneity analysis revealed no significant heterogeneity, with I^2^ = 0%. Tests for heterogeneity within designs were not significant (Q = 6.76, df = 10, *p* = 0.7481), indicating consistency among the study results. The P-scores, which rank treatments based on their likelihood of causing diarrhea, were highest for GLP-1 (0.41), followed by Resmetirom (0.27), and FGF21 (0.20). There was no difference in the subgroups (Supplement S10) in the Bayesian analysis, indicating the robustness of the results.

## 4. Discussion

The findings of this network meta-analysis reveal significant clinical insights into the efficacy of FGF21 analogs, GLP-1 agonists, and Resmetirom for treating liver conditions. FGF21 analogs showed the highest effectiveness in terms of MASH resolution and fibrosis improvement. Resmetirom also demonstrated substantial efficacy, particularly in reducing liver fat content and improving ALT and AST levels, highlighting its potential in managing liver fat and inflammation. GLP-1 agonists, while effective in reducing liver fat and improving AST levels, were less impactful on fibrosis. However, both Resmetirom and GLP-1 agonists showed significant reductions in ALT and GGT levels. Adverse events analysis indicated that Resmetirom and FGF21 analogs were associated with higher risks of nausea and diarrhea.

To our knowledge, this study is the first to provide a practical comparison of FGF21 analogs, Resmetirom, and GLP-1 agonist treatments for clinical practice, highlighting the significance of the efficacy and safety data. Our analysis provides a detailed assessment of different outcomes, including fibrosis improvement, liver fat reduction, and changes in biochemical markers such as ALT, AST, and GGT. By providing information about adverse events, treatment discontinuation due to adverse events, and some highlights of the common adverse events, these findings underscore the importance of considering adverse event profiles and treatment discontinuation rates when selecting a therapeutic regimen.

The clinical outcomes observed are closely linked to the mechanisms of action of Resmetirom, FGF21 analogs, and GLP-1 agonists. Resmetirom, a selective thyroid hormone receptor-β agonist, accelerates hepatic fat metabolism, demonstrating substantial efficacy in reducing liver fat (MRI-PDFF) and improving liver enzymes (ALT, AST). This highlights Resmetirom’s effectiveness in targeting liver fat accumulation and enhancing liver function [4]. FGF21 analogs’ anti-inflammatory properties help reduce liver inflammation and fibrosis [5], which could explain their effectiveness in fibrosis improvement and MASH resolution. GLP-1 agonists primarily work by enhancing insulin secretion, inhibiting glucagon release, promoting weight loss, and improving lipid profiles [6]. This explains their effectiveness in reducing liver fat and improving liver enzymes, although their impact on fibrosis is less pronounced. The adverse events associated with these medications, such as nausea and diarrhea, are consistent with their metabolic and gastrointestinal effects, reflecting their broad systemic actions.

Furthermore, this study highlights the need for long-term follow-up and real-world evidence to validate the clinical benefits and safety profiles of these treatments. The observed heterogeneity across studies emphasizes the importance of individualized patient care, as treatment responses may vary due to genetic, metabolic, and lifestyle factors. Additionally, the high costs of therapies, for example, the annual cost of Resmetirom being around $47,700 [33], necessitate cost-effectiveness analyses to determine their value in routine practice. Future research should focus on head-to-head comparisons, combination therapy strategies, and integrating these agents into clinical guidelines for MASLD and MASH management. These treatments’ potential to improve liver health and address comorbid conditions like obesity and diabetes supports their inclusion in a comprehensive treatment approach.

At this time, GLP-1 agonists and FGF21 analogs are not approved for the treatment of MASH/MASLD. These agents are still under investigation in ongoing clinical trials, though early results are promising. For example, the ESSENCE trial is a Phase 3 study evaluating the use of semaglutide, a GLP-1 receptor agonist, in approximately 1200 patients with NASH. The primary endpoint of this study is to assess the efficacy of a weekly 2.4 mg dose of semaglutide in improving NASH without worsening fibrosis after 72 weeks. Pegozafermin, an FGF21 analog, is being evaluated in the ENLIGHTEN program. This includes the ENLIGHTEN-Fibrosis trial, which targets patients with F2-F3 fibrosis, and the ENLIGHTEN-Cirrhosis trial, aimed at patients with compensated cirrhosis (F4). Both trials have co-primary endpoints focused on fibrosis improvement and MASH resolution, aiming for future regulatory approval [34,35].

This study, while comprehensive, faces several limitations. Key challenges include variability across studies in populations, interventions, and outcome measures, introducing heterogeneity and complicating direct comparisons. Differences in the quality and methodological rigor of the studies potentially affect the robustness of pooled estimates. Reliance on published data may lead to publication bias, and the lack of individual patient data restricts detailed subgroup analyses. Variations in study designs, follow-up durations, and outcome definitions add complexity, particularly given the low certainty of evidence for most comparisons. Additionally, it must be noted that this analysis compares the only approved drug for this condition against two other classes of agents, GLP-1 agonists and FGF21 analogs, which are still under investigation. This inherently adds heterogeneity to the comparisons and assumes a similar effect across all studied GLP-1 drugs and FGF21 agents, which may not fully capture the nuances in their individual efficacy and safety profiles. Furthermore, a key limitation is that the included trials were conducted using the diagnostic criteria for NAFLD, whereas the newer classification, MASLD, emphasizes metabolic dysfunction more clearly. While MASLD reclassifies NAFLD with a focus on metabolic drivers, the therapeutic interventions studied, such as GLP-1 agonists and Resmetirom, target shared underlying mechanisms, including insulin resistance, lipid metabolism, and inflammation. Therefore, the results and conclusions from these NAFLD-based trials remain relevant to MASLD populations. However, the shift in diagnostic criteria could influence patient selection in future studies, potentially affecting generalizability. Further research using MASLD-specific criteria may provide more precise insights into this population.

## Figures and Tables

**Table 1:** Study characteristics and outcomes in the included clinical trials.

Study	Design	Registration	Duration	Treatment Arms	Primary Outcomes	Secondary Outcomes	Population
Abdelmalek, 2024 [6]	Phase2b, RCT, DB, PC	NCT04031729	24 weeks	Pegbelfermin (10 mg, 20 mg, 40 mg weekly), placebo	Improvement in fibrosis without worsening of NASH	NAFLD activity score, liver variables, metabolic variables, safety	Patients with NASH, aged 21–75 years, fibrosis stage F2/F3, NAFLD score ≥4
Armstrong, 2016 [19]	RCT, DB, PC	NCT01237119	48 weeks	Liraglutide (1.8 mg daily), placebo	Resolution of NASH without worsening of fibrosis	Changes in NAFLD activity score, liver enzymes, metabolic parameters, quality of life	Patients with NASH, aged 18–75 years, NAFLD activity score ≥4
Flint, 2021 [20]	RCT, DB, PC	NCT03486899	48 weeks	Semaglutide (0.4 mg daily), placebo	Change in liver stiffness (MRE)	Changes in liver fat content, liver enzymes, glucose metabolism, cardiovascular risk factors, safety	Patients with NASH, aged 18–75 years, liver stiffness by MRE ≥3.64 kPa
Guo, 2020 [32]	RCT, PC	ChiCTR2000035091	26 weeks	Placebo, insulin glargine, liraglutide	Changes in IHCL, abdominal adiposity (SAT and VAT)	Changes in liver function (AST, ALT), glycemia (HbA1c, FPG), body weight, BMI	Adults with T2D and NAFLD
Harrison, 2021 [21]	RCT, DB, PC, phase 2a	NCT03976401	16 weeks	Placebo, efruxifermin 28 mg, 50 mg, 70 mg	Absolute change in hepatic fat fraction (HFF)	Percent change in HFF, responders, change in ALT, safety and tolerability	Adults with biopsy-proven NASH
Harrison, 2023 [26]	RCT, DB, PC, phase IIa	NCT03976401	26 weeks	Placebo, efruxifermin 50 mg weekly	Safety, tolerability	Change in liver stiffness, non-invasive biomarkers of fibrosis, liver histopathology, markers of liver injury and metabolism	NASH with compensated cirrhosis
Harrison, 2023 [25]	RCT, DB, PC, phase 2b	NCT04767529	24 weeks	Placebo, efruxifermin 28 mg weekly, 50 mg weekly	Improvement in liver fibrosis by ≥1 stage without worsening of NASH	NASH resolution, change in HFF by MRI-PDFF, non-invasive markers of fibrosis, glycaemic control, lipid metabolism, safety, tolerability, immunogenicity	Adults with NASH and fibrosis stages 2–3
Kuchay, 2018 [30]	RCT, open-label, controlled	NCT02686476	20 weeks	Control, empagliflozin 10 mg daily	Change in liver fat content (MRI-PDFF)	Changes in AST, ALT, GGT levels	Adults with T2D and NAFLD
Loomba, 2023 [22]	RCT, DB, PC, phase 1b/2a	NCT04048135	12 weeks	Placebo, pegozafermin 3 mg, 9 mg, 18 mg weekly, 27 mg weekly, 18 mg biweekly, 36 mg biweekly	Safety, tolerability, pharmacokinetics	Changes in hepatic fat fraction (MRI-PDFF), bodyweight, lipid profile, liver enzymes, immunogenicity	Adults with NASH
Loomba, 2023 [28]	Multinational, RCT, DB, PC, phase 2b	NCT04929483	24 weeks	Pegozafermin (15 mg, 30 mg weekly, 44 mg biweekly), placebo	Improvement in liver fibrosis, NASH resolution	NAFLD activity score, liver variables, metabolic variables, safety	Patients with NASH, aged 21–75 years, fibrosis stage F2/F3, NAFLD score ≥4
Loomba, 2023 [27]	RCT, DB, PC phase 2 trial	NCT03987451	48 weeks	Semaglutide 2.4 mg once weekly vs. placebo	Improvement in liver fibrosis without worsening NASH	Liver fat content change (MRI-PDFF), NASH resolution, fibrosis stage change, adverse events	Biopsy-confirmed NASH-related cirrhosis, BMI ≥27 kg/m^2^
Newsome, 2021 [29]	RCT, DB, PC	NCT02970942	72 weeks	Semaglutide (0.1 mg, 0.2 mg, 0.4 mg daily), placebo	Resolution of NASH without worsening of fibrosis	Changes in fibrosis stage, liver enzymes, metabolic parameters, safety	Patients with NASH, aged 18–75 years, fibrosis stage F1-F3, NAFLD score ≥4
Sanyal, 2018 [31]	RCT, DB, PC, phase 2a	NCT02413372	16 weeks	Placebo, pegbelfermin 10 mg daily, 20 mg weekly	Safety, tolerability, hepatic fat fraction change	Pharmacokinetics, immunogenicity, exploratory endpoints	NASH patients
Harrison, 2019 [4]	DB, RCT, PC	NCT03987451	36 weeks	Resmetirom 80 mg, placebo	Percent relative change in hepatic fat fraction by MRI-PDFF at 12 weeks	Proportions of patients with ≥30% hepatic fat reduction at 12 and 36 weeks; absolute hepatic fat reduction at 12 and 36 weeks; changes in liver enzymes, fibrosis biomarkers, and lipids	Adults with biopsy-confirmed NASH; ≥18 years; ≥10% hepatic fat on screening MRI-PDFF
Harrison, 2023 [24]	RCT, DB, PC, phase 3	NCT04197479	52 weeks	Resmetirom 100 mg OL, resmetirom 100 mg DB, resmetirom 80 mg DB, placebo DB	Safety and tolerability of resmetirom in patients with NAFLD (presumed NASH)	Proportion of patients achieving ≥30% reduction in liver fat content (MRI-PDFF); changes in liver volume, liver fat volume, VAT, SAT, body weight, waist circumference, BMI, liver enzymes, glucose metabolism, cardiovascular risk factors, and exploratory blood biomarkers	Adults ≥18 years with ≥3 metabolic risk factors; Patients with NAFLD (presumed NASH); Acceptable standard blood chemistry and hematology results; ≥8% hepatic fat (MRI-PDFF)
Harrison, 2024 [23]	RCT, DB, PC, phase 3	NCT03900429	52 weeks	Resmetirom 80 mg, resmetirom 100 mg, placebo	≥2 point reduction in NAFLD activity score without worsening fibrosis. Fibrosis improvement: ≥1 stage increase without worsening NAFLD activity score	Change in LDL cholesterol at week 24; changes in liver enzymes and noninvasive tests	Adults with biopsy-confirmed NASH and fibrosis stages F1B, F2, or F3; 966 patients

The table summarizes various NASH clinical trials, detailing study design, registration, duration, treatment arms, primary and secondary outcomes, and population characteristics. Abbreviations used include RCT (randomized controlled trial), DB (double-blind), PC (placebo-controlled), NAFLD (non-alcoholic fatty liver disease), NASH (non-alcoholic steatohepatitis), and MRI-PDFF (magnetic resonance imaging-proton density fat fraction). Abbreviations: RCT: Randomized Controlled Trial; DB: Double-Blind; PC: Placebo-Controlled; NAFLD: Non-Alcoholic Fatty Liver Disease; NASH: Non-Alcoholic Steatohepatitis; MRI-PDFF: Magnetic Resonance Imaging-Proton Density Fat Fraction; IHCL: Intrahepatocellular Lipid; SAT: Subcutaneous Adipose Tissue; VAT: Visceral Adipose Tissue; MRE: Magnetic Resonance Elastography; HFF: Hepatic Fat Fraction; ALT: Alanine Aminotransferase; AST: Aspartate Aminotransferase; GGT: Gamma-Glutamyl Transferase; BMI: Body Mass Index; FPG: Fasting Plasma Glucose; HbA1c: Hemoglobin A1c.

**Table 2:** Baseline characteristics of patients.

Study	Participants	Age (years)	Sex (Male) (%)	BMI (kg/m^2^)	ALT (U/L)	AST (U/L)	HbA1c (%)
Abdelmalek, 2024 [6]	154	59.4(8.7)	36.0	35.6(6.1)	48.6(26.3)	45.5(24.1)	6.9(1.1)
Armstrong, 2016 [19]	52	51.0(11.5)	60.0	35.9(5.5)	71.5(38.0)	51.0(24.5)	6.0(0.8)
Flint, 2021 [20]	67	60.0(9.3)	65.0	35.4(5.9)	37.5(83.7)	30.0(67.2)	7.4(1.0)
Guo, 2020 [32]	91	57.1(11.2)	46.0	34.6(7.5)	54.4(28.6)	29.5(16.3)	6.7(1.3)
Harrison, 2019 [4]	125	50.2(11.5)	51.0	35.1(6.1)	52.6(30.8)	37.2(18.6)	6.3(1.1)
Harrison, 2021 [21]	80	54.3(12.0)	48.0	37.7(6.8)	51.5(30.0)	37.4(17.4)	6.6(1.2)
Harrison, 2023 [24]	1185	55.8(11.8)	51.8	35.5(6.1)	37.0(25.4)	25.7(14.1)	6.0(0.0)
Harrison, 2023 [26]	30	51.1(11.6)	41.0	38.4(8.1)	58.6(29.2)	40.3(18.4)	6.3(1.0)
Harrison, 2023 [25]	128	52.7(13.0)	38.1	37.5(7.3)	37.0(13.8)	37.0(13.8)	6.7(1.1)
Harrison, 2024 [23]	966	56.7(11.0)	55.8	35.7(6.8)	54.6(32.0)	40.4(23.0)	-
Kuchay, 2018 [30]	42	52.3(6.9)	-	29.7(3.5)	56.8(30.3)	44.9(23.9)	9.0(1.1)
Loomba, 2023 [22]	81	51.9(9.8)	38.5	34.6(4.8)	55.4(39.2)	30.9(20.7)	9.0(1.1)
Loomba, 2023 [28]	71	59.2(8.2)	30.0	35.0(5.9)	44.5(58.2)	44.4(45.8)	7.2(1.3)
Loomba, 2023 [27]	71	55.5(10.5)	34.0	36.8(5.6)	56.8(30.6)	44.0(23.0)	6.8(1.2)
Newsome, 2021 [29]	320	55.0(10.5)	58.0	35.8(6.4)	54.0(86.0)	43.0(79.0)	7.3(1.2)
Sanyal, 2018 [31]	75	50.3(11.6)	35.8	35.4(5.6)	42.5(22.4)	53.5(33.4)	6.1(1.0)

The table summarizes the baseline characteristics of patients included in the study. Characteristics include the number of participants, age, male participants, body mass index (BMI), levels of alanine aminotransferase (ALT) and aspartate aminotransferase (AST), and glycated hemoglobin (HbA1c). Mean values with SD are provided for continuous variables, while percentages are provided for categorical variables. Abbreviations: BMI: Body mass index; ALT: Alanine aminotransferase; AST: Aspartate aminotransferase; HbA1c: Glycated hemoglobin.

## Data Availability

All original contributions presented in this study are included within the submitted material. For additional inquiries, please contact the corresponding author.

## Supplementary Materials

The following supporting information can be downloaded at: https://www.mdpi.com/article/10.3390/biomedicines12102328/s1, References [36,37,38,39,40,41,42,43] are cited in the supplementary materials. Supplement S1: Search strategy. Supplement S2: Baseline characteristics of the included studies. Supplement S3: Baseline Characteristics of the participants. Supplement S4: Risk of bias assessment of included trials for each outcome. Supplement S5: Network plots of treatment comparisons. Supplement 6: Publication bias (funnel plot). Supplement S7: Certainty of the effect estimates. Supplement S8: Certainty of the effect estimates. Supplement S9: Summary of the excluded studies. Supplement S10: Meta-regression. Supplement S11: PRISMA checklist.

## References

[1] Targher G., Byrne C.D., Tilg H. (2024). MASLD: A systemic metabolic disorder with cardiovascular and malignant complications. Gut.

[2] Sanyal A.J., Husain M., Diab C., Mangla K.K., Shoeb A., Lingvay I., Tapper E.B. (2024). Cardiovascular disease in patients with metabolic dysfunction-associated steatohepatitis compared with metabolic dysfunction-associated steatotic liver disease and other liver diseases: A systematic review. Am. Heart J. Plus.

[3] Younossi Z.M., Koenig A.B., Abdelatif D., Fazel Y., Henry L., Wymer M. (2016). Global epidemiology of nonalcoholic fatty liver disease-Meta-analytic assessment of prevalence, incidence, and outcomes. Hepatology.

[4] Harrison S.A., Bashir M.R., Guy C.D., Zhou R., A Moylan C., Frias J.P., Alkhouri N., Bansal M.B., Baum S., A Neuschwander-Tetri B. (2019). Resmetirom (MGL-3196) for the treatment of non-alcoholic steatohepatitis: A multicentre, randomised, double-blind, placebo-controlled, phase 2 trial. Lancet.

[5] Kliewer S.A., Mangelsdorf D.J. (2019). A Dozen Years of Discovery: Insights into the Physiology and Pharmacology of FGF21. Cell Metab..

[6] Abdelmalek M.F., Harrison S.A., Sanyal A.J. (2024). The role of glucagon-like peptide-1 receptor agonists in metabolic dysfunction-associated steatohepatitis. Diabetes Obes. Metab..

[7] Ayesh H. (2024). Comparative Analysis of Resmetirom vs FGF21 Agonists vs. GLP-1 Agonists in MASLD and MASH: Network Meta-Analysis of Clinical Trials. https://osf.io/hxvp3.

[8] Page M.J., McKenzie J.E., Bossuyt P.M., Boutron I., Hoffmann T.C., Mulrow C.D., Shamseer L., Tetzlaff J.M., Akl E.A., Brennan S.E. (2021). The PRISMA 2020 statement: An updated guideline for reporting systematic reviews. BMJ.

[9] Wan X., Wang W., Liu J., Tong T. (2014). Estimating the sample mean and standard deviation from the sample size, median, range and/or interquartile range. BMC Med. Res. Methodol..

[10] Higgins J.P.T., Thomas J., Chandler J., Welch V., Higgins J.P., Thomas J. (2019). Cochrane Handbook for Systematic Reviews of Interventions, Version 6.3 ed.

[11] Rouse B., Chaimani A., Li T. (2017). Network meta-analysis: An introduction for clinicians. Intern. Emerg. Med..

[12] Higgins J.P., Thompson S.G., Deeks J.J., Altman D.G. (2003). Measuring inconsistency in meta-analyses. BMJ.

[13] Allaire J. (2023). RStudio: Integrated Development Environment for R.

[14] Balduzzi S., Rücker G., Nikolakopoulou A., Papakonstantinou T., Salanti G., Efthimiou O., Schwarzer G. (2023). netmeta: An R Package for Network Meta-Analysis Using Frequentist Methods. J. Stat. Softw..

[15] Rücker G., Schwarzer G. (2015). Ranking treatments in frequentist network meta-analysis works without resampling methods. BMC Med. Res. Methodol..

[16] Sterne J.A.C., Savović J., Page M.J., Elbers R.G., Blencowe N.S., Boutron I., Cates C.J., Cheng H.Y., Corbett M.S., Eldridge S.M. (2019). RoB 2: A revised tool for assessing risk of bias in randomised trials. BMJ.

[17] Nikolakopoulou A., Higgins J.P.T., Papakonstantinou T., Chaimani A., Del Giovane C., Egger M., Salanti G. (2020). CINeMA: An approach for assessing confidence in the results of a network meta-analysis. PLoS Med..

[18] Abdelmalek M.F., Charles E.D., Sanyal A.J., Harrison S.A., Neuschwander-Tetri B.A., Goodman Z., Ehman R.A., Karsdal M., Nakajima A., Du S. (2021). The FALCON program: Two phase 2b randomized, double-blind, placebo-controlled studies to assess the efficacy and safety of pegbelfermin in the treatment of patients with nonalcoholic steatohepatitis and bridging fibrosis or compensated cirrhosis. Contemp. Clin. Trials.

[19] Armstrong M.J., Gaunt P., Aithal G.P., Barton D., Hull D., Parker R., Hazlehurst J.M., Guo K., Abouda G., A Aldersley M. (2016). Liraglutide safety and efficacy in patients with non-alcoholic steatohepatitis (LEAN): A multicentre, double-blind, randomised, placebo-controlled phase 2 study. Lancet.

[20] Flint A., Andersen G., Hockings P., Johansson L., Morsing A., Palle M.S., Vogl T., Loomba R., Plum-Mörschel L. (2021). Randomised clinical trial: Semaglutide versus placebo reduced liver steatosis but not liver stiffness in subjects with non-alcoholic fatty liver disease assessed by magnetic resonance imaging. Aliment. Pharmacol. Ther..

[21] Harrison S.A., Ruane P.J., Freilich B.L., Neff G., Patil R., Behling C.A., Hu C., Fong E., de Temple B., Tillman E.J. (2021). Efruxifermin in non-alcoholic steatohepatitis: A randomized, double-blind, placebo-controlled, phase 2a trial. Nat. Med..

[22] Loomba R., Lawitz E.J., Frias J.P., Ortiz-Lasanta G., Johansson L., Franey B.B., Morrow L., Rosenstock M., Hartsfield C.L., Chen C.-Y. (2023). Safety, pharmacokinetics, and pharmacodynamics of pegozafermin in patients with non-alcoholic steatohepatitis: A randomised, double-blind, placebo-controlled, phase 1b/2a multiple-ascending-dose study. Lancet Gastroenterol. Hepatol..

[23] Harrison S.A., Bedossa P., Guy C.D., Schattenberg J.M., Loomba R., Taub R., Labriola D., Moussa S.E., Neff G.W., Rinella M.E. (2024). A Phase 3, Randomized, Controlled Trial of Resmetirom in NASH with Liver Fibrosis. N. Engl. J. Med..

[24] Harrison S.A., Taub R., Neff G.W., Lucas K.J., Labriola D., Moussa S.E., Alkhouri N., Bashir M.R. (2023). Resmetirom for nonalcoholic fatty liver disease: A randomized, double-blind, placebo-controlled phase 3 trial. Nat. Med..

[25] Harrison S.A., Ruane P.J., Freilich B., Neff G., Patil R., Behling C., Hu C., Shringarpure R., de Temple B., Fong E. (2023). A randomized, double-blind, placebo-controlled phase IIa trial of efruxifermin for patients with compensated NASH cirrhosis. JHEP Rep..

[26] Harrison S.A., Frias J.P., Neff G., A Abrams G., Lucas K.J., Sanchez W., Gogia S., Sheikh M.Y., Behling C., Bedossa P. (2023). Safety and efficacy of once-weekly efruxifermin versus placebo in non-alcoholic steatohepatitis (HARMONY): A multicentre, randomised, double-blind, placebo-controlled, phase 2b trial. Lancet Gastroenterol. Hepatol..

[27] Loomba R., Abdelmalek M.F., Armstrong M.J., Jara M., Kjær M.S., Krarup N., Lawitz E., Ratziu V., Sanyal A.J., Schattenberg J.M. (2023). Semaglutide 2·4 mg once weekly in patients with non-alcoholic steatohepatitis-related cirrhosis: A randomised, placebo-controlled phase 2 trial. Lancet Gastroenterol. Hepatol..

[28] Loomba R., Sanyal A.J., Kowdley K.V., Bhatt D.L., Alkhouri N., Frias J.P., Bedossa P., Harrison S.A., Lazas D., Barish R. (2023). Randomized, Controlled Trial of the FGF21 Analogue Pegozafermin in NASH. N. Engl. J. Med..

[29] Newsome P.N., Buchholtz K., Cusi K., Linder M., Okanoue T., Ratziu V., Sanyal A.J., Sejling A.-S., Harrison S.A. (2021). A Placebo-Controlled Trial of Subcutaneous Semaglutide in Nonalcoholic Steatohepatitis. N. Engl. J. Med..

[30] Kuchay M.S., Krishan S., Mishra S.K., Farooqui K.J., Singh M.K., Wasir J.S., Bansal B., Kaur P., Jevalikar G., Gill H.K. (2018). Effect of Empagliflozin on Liver Fat in Patients with Type 2 Diabetes and Nonalcoholic Fatty Liver Disease: A Randomized Controlled Trial (E-LIFT Trial). Diabetes Care.

[31] Sanyal A., Charles E.D., Neuschwander-Tetri B.A., Loomba R., A Harrison S., Abdelmalek M.F., Lawitz E.J., Halegoua-DeMarzio D., Kundu S., Noviello S. (2019). Pegbelfermin (BMS-986036), a PEGylated fibroblast growth factor 21 analogue, in patients with non-alcoholic steatohepatitis: A randomised, double-blind, placebo-controlled, phase 2a trial. Lancet.

[32] Guo W., Tian W., Lin L., Xu X. (2020). Liraglutide or insulin glargine treatments improves hepatic fat in obese patients with type 2 diabetes and nonalcoholic fatty liver disease in twenty-six weeks: A randomized placebo-controlled trial. Diabetes Res. Clin. Pract..

[33] Leo L., Sunny M.E. US FDA Approves First Drug for Fatty Liver Disease NASH. Reuters. https://www.reuters.com/business/healthcare-pharmaceuticals/us-fda-approves-first-drug-fatty-liver-disease-nash-2024-03-14.

[34] Bio I. 89bio Initiates Phase 3 ENLIGHTEN-Fibrosis Trial of Pegozafermin in Non-Cirrhotic Metabolic Dysfunction-Associated Steatohepatitis (MASH) Patients with Fibrosis. https://ir.89bio.com/news-releases/news-release-details/89bio-initiates-phase-3-enlighten-fibrosis-trial-pegozafermin.

[35] ClinicalTrials.gov A Study of Semaglutide in Participants with Nonalcoholic Steatohepatitis (ESSENCE). 4 October 2024. https://clinicaltrials.gov/study/NCT04822181?cond=NCT04822181&rank=1.

[36] Harrison S.A., Alkhouri N., Davison B.A., Sanyal A., Edwards C., Colca J.R., Lee B.H., Loomba R., Cusi K., Kolterman O. (2020). Insulin sensitizer MSDC-0602K in non-alcoholic steatohepatitis: A randomized, double-blind, placebo-controlled phase IIb study. J. Hepatol..

[37] Ratziu V., Harrison S.A., Francque S., Bedossa P., Lehert P., Serfaty L., Romero-Gomez M., Boursier J., Abdelmalek M., Caldwell S. (2016). Elafibranor, an Agonist of the Peroxisome Proliferator-Activated Receptor-α and -δ, Induces Resolution of Nonalcoholic Steatohepatitis Without Fibrosis Worsening. Gastroenterology.

[38] Khoo J., Hsiang J.C., Taneja R., Koo S.H., Soon G.H., Kam C.J., Law N.M., Ang T.L. (2019). Randomized trial comparing effects of weight loss by liraglutide with lifestyle modification in non-alcoholic fatty liver disease. Liver Int..

[39] Metzner V., Herzog G., Heckel T., Bischler T., Hasinger J., Otto C., Fassnacht M., Geier A., Seyfried F., Dischinger U. (2022). Liraglutide + PYY(3-36) Combination Therapy Mimics Effects of Roux-en-Y Bypass on Early NAFLD Whilst Lacking-Behind in Metabolic Improvements. J. Clin. Med..

[40] Javanbakht M., Fishman J., Moloney E., Rydqvist P., Ansaripour A. (2023). Early Cost-Effectiveness and Price Threshold Analyses of Resmetirom: An Investigational Treatment for Management of Nonalcoholic Steatohepatitis. Pharmacoecon Open.

[41] Brown E.A., Minnich A., Sanyal A.J., Loomba R., Du S., Schwarz J., Ehman R.L., Karsdal M., Leeming D.J., Cizza G. (2023). Effect of pegbelfermin on NASH and fibrosis-related biomarkers and correlation with histological response in the FALCON 1 trial. JHEP Rep..

[42] Lu Y., Yu B., Bu Y., Lou J., Jin Y. (2024). Pegbelfermin for reducing transaminase levels in patients with non-alcoholic steatohepatitis: A dose-response meta-analysis of randomized controlled trials. Front. Med..

[43] Zhao Y., Zhao W., Bu H., Toshiyoshi M., Zhao Y. (2023). Liraglutide on type 2 diabetes mellitus with nonalcoholic fatty liver disease: A systematic review and meta-analysis of 16 RCTs. Medicine.

