# Supplementary material for: Comparative Analysis of Resmetirom vs. FGF21 Analogs vs. GLP-1 Agonists in MASLD and MASH: Network Meta-Analysis of Clinical Trials
Source: Biomedicines. 2024 Oct 14;12(10):2328. doi: 10.3390/biomedicines12102328 (PMC11505228; doi:10.3390/biomedicines12102328)
Supplement: Supplementary file 1 [file biomedicines-12-02328-s001.zip › biomedicines-3226282-supplementary.pdf]

Table of Contents

Supplement S1. Search strategy 4

Supplement S2. Baseline characteristics of the included studies 5

Abbreviations: 7

Supplement S3. Baseline Characteristics of the participants 8

Supplement S4. Risk of bias assessment of included trials for each outcome 9

4.1 NASH resolution 9

4.2 Improvement in Fibrosis 10

4.3 Hepatic fat reduction (MRI PDFF) 10

4.4 >30% Fat Reduction on MRI-PDFF 11

4.5 Change in VCTE 11

4.6 Change in ALT 12

4.7 Change in AST 12

4.8 Change in GGT 12

4.9 Adverse events 13

4.10 Treatment discontinuation 14

4.11 Nausea 15

4.12 Diarrhea 16

Supplement S5. Summary of the excluded studies: 17

Figure S5.1. Network Plot of Treatment Comparisons for NASH Resolution 17

Figure S5.2. Network Plot of Treatment Comparisons for improvement in fibrosis 17

Figure S5.3. Network Plot of Treatment Comparisons for change in MRI-PDFF 18

Figure S5.4. Network Plot of Treatment Comparisons for >30% Fat Reduction on MRI-PDFF 18

Figure S5.5. Network Plot of Treatment Comparisons for Change in VCTE 19

Figure S5.6. Network Plot of Treatment Comparisons for change in ALT 19

Figure S5.7. Network Plot of Treatment Comparisons for change in AST 20

Figure S5.8. Network Plot of Treatment Comparisons for change in GGT 20

Figure S5.9. Network Plot of Treatment Comparisons for adverse events 21

Figure S5.10. Network Plot of Treatment Comparisons for treatment discontinuation 21

Figure S5.11. Network Plot of Treatment Comparisons for nausea 22

Figure S5.12. Network Plot of Treatment Comparisons for diarrhea 22

Supplement S6. Publication bias (funnel plot) 23

6.1 NASH resolution 23

6.2 Improvement in Fibrosis 23

6.3 Hepatic fat reduction (MRI PDFF) 23

6.4 >30% Fat Reduction on MRI-PDFF 24
6.5 Change in VCTE 24
6.6 Change in ALT 24
6.7 Change in AST 25
6.8 Change in GGT 25
6.9 Adverse events 25
6.10 Treatment discontinuation 26
6.11 Nausea 26
6.12 Diarrhea 26
Supplement S7. Certainty of the effect estimates 27
7.1 NASH resolution 27
7.2 Improvement in Fibrosis 27
7.3 Hepatic fat reduction (MRI PDFF) 27
7.4 >30% Fat Reduction on MRI-PDFF 28
7.5 Change in VCTE 28
7.6 Change in ALT 28
7.7 Change in AST 28
7.8 Change in GGT 28
7.9 Adverse events 29
7.10 Treatment discontinuation 29
7.11 Nausea 29
7.12 Diarrhea 29
Supplement S8. Certainty of the effect estimates 30
8.1 NASH resolution 30
8.2 Improvement in Fibrosis 30
8.3 Hepatic fat reduction (MRI PDFF) 31
8.4 >30% Fat Reduction on MRI-PDFF 31
8.5 Change in VCTE 32
8.6 Change in ALT 32
8.7 Change in AST 33
8.8 Change in GGT 33
8.9 Adverse events 34
8.10 Treatment discontinuation 34
8.11 Nausea 35
8.12 Diarrhea 35
Supplement S9: Summary of the excluded studies: 36

Supplement S10: Meta-regression: *37*

10.1 NASH resolution **37**

10.2 Improvement in Fibrosis **38**

10.3 Hepatic fat reduction (MRI PDFF) **39**

10.4 >30% Fat Reduction on MRI-PDFF **40**

10.5 Change in VCTE **41**

10.6 Change in ALT **42**

10.7 Change in AST **43**

10.8 Change in GGT **44**

10.9 Adverse events **45**

10.10 Treatment discontinuation **46**

10.11 Nausea **47**

10.12 Diarrhea **48**

Supplement S11. PRISMA checklist *49*

References *53*

Supplement S1. Search strategy

From inception to June, 8th 2024:

Pubmed
("Resmetirom" OR "MGL-3196" OR "Thyroid hormone receptor-β agonist") OR ("FGF21 analogs" OR "Pegozafermin" OR "BIO89-100" OR "Pegbelfermin" OR "BMS-986036" OR "Efruxifermin" OR "AKR-001") OR ("GLP-1 agonists" OR "liraglutide" OR "semaglutide" OR "dulaglutide") AND ("MASLD" OR "MASH" OR "NASH" OR "MAFLD" OR "NAFLD") AND ("clinical trials" OR "randomized controlled trials" OR "RCTs" OR "trial")

Web of Science
("Resmetirom" OR "MGL-3196" OR "Thyroid hormone receptor-β agonist") OR ("FGF21 analogs" OR "Pegozafermin" OR "BIO89-100" OR "Pegbelfermin" OR "BMS-986036" OR "Efruxifermin" OR "AKR-001") OR ("GLP-1 agonists" OR "liraglutide" OR "semaglutide" OR "dulaglutide") AND ("MASLD" OR "MASH" OR "NASH" OR "MAFLD" OR "NAFLD") AND ("clinical trials" OR "randomized controlled trials" OR "RCTs" OR "trial")

Scopus
("Resmetirom" OR "MGL-3196" OR "Thyroid hormone receptor-β agonist") OR ("FGF21 analogs" OR "Pegozafermin" OR "BIO89-100" OR "Pegbelfermin" OR "BMS-986036" OR "Efruxifermin" OR "AKR-001") OR ("GLP-1 agonists" OR "liraglutide" OR "semaglutide" OR "dulaglutide") AND ("MASLD" OR "MASH" OR "NASH" OR "MAFLD" OR "NAFLD") AND ("clinical trials" OR "randomized controlled trials" OR "RCTs" OR "trial")

Cochrane
("Resmetirom" OR "MGL-3196" OR "Thyroid hormone receptor-β agonist") OR ("FGF21 analogs" OR "Pegozafermin" OR "BIO89-100" OR "Pegbelfermin" OR "BMS-986036" OR "Efruxifermin" OR "AKR-001") OR ("GLP-1 agonists" OR "liraglutide" OR "semaglutide" OR "dulaglutide") AND ("MASLD" OR "MASH" OR "NASH" OR "MAFLD" OR "NAFLD") AND ("clinical trials" OR "randomized controlled trials" OR "RCTs" OR

Supplement S2. Baseline characteristics of the included studies
Table S2: Study Characteristics and Outcomes in the included Clinical Trials

The table summarizes various NASH clinical trials, detailing study design, registration, duration, treatment arms, primary and secondary outcomes, and population characteristics. Abbreviations used include RCT (randomized controlled trial), DB (double-blind), PC (placebo-controlled), NAFLD (non-alcoholic fatty liver disease), NASH (non-alcoholic steatohepatitis), and MRI-PDFF (magnetic resonance imaging-proton density fat fraction).

Study	Design	Registration	Duration	Treatment Arms	Primary Outcomes	Secondary Outcomes	Population
**Abdelmalek, 2024**	Phase2b, RCT, DB, PC	NCT04031729	24 weeks	Pegbelfermin (10 mg, 20 mg, 40 mg weekly), Placebo	Improvement in fibrosis without worsening of NASH	NAFLD activity score, liver variables, metabolic variables, safety	Patients with NASH, aged 21–75 years, fibrosis stage F2/F3, NAFLD score ≥4
**Armstrong, 2016**	RCT, DB, PC	NCT01237119	48 weeks	Liraglutide (1.8 mg daily), Placebo	Resolution of NASH without worsening of fibrosis	Changes in NAFLD activity score, liver enzymes, metabolic parameters, quality of life	Patients with NASH, aged 18–75 years, NAFLD activity score ≥4
**Flint, 2021**	RCT, DB, PC	NCT03486899	48 weeks	Semaglutide (0.4 mg daily), Placebo	Change in liver stiffness (MRE)	Changes in liver fat content, liver enzymes, glucose metabolism, cardiovascular risk factors, safety	Patients with NASH, aged 18–75 years, liver stiffness by MRE ≥3.64 kPa
**Guo, 2020**	RCT, PC	ChiCTR2000035091	26 weeks	Placebo, Insulin glargine, Liraglutide	Changes in IHCL, abdominal adiposity (SAT and VAT)	Changes in liver function (AST, ALT), glycemia (HbA1c, FPG), body weight, BMI	Adults with T2D and NAFLD
**Harrison, 2021**	RCT, DB, PC, phase 2a	NCT03976401	16 weeks	Placebo, Efruxifermin 28 mg, 50 mg, 70 mg	Absolute change in hepatic fat fraction (HFF)	Percent change in HFF, responders, change in ALT, safety and tolerability	Adults with biopsy-proven NASH
**Harrison, 2023_b**	RCT, DB, PC, phase IIa	NCT03976401	26 weeks	Placebo, Efruxifermin 50 mg weekly	Safety, tolerability	Change in liver stiffness, non-invasive biomarkers of fibrosis, liver histopathology, markers of liver injury and metabolism	NASH with compensated cirrhosis
**Harrison, 2023_c**	RCT, DB, PC, phase 2b	NCT04767529	24 weeks	Placebo, Efruxifermin 28 mg weekly, 50 mg weekly	Improvement in liver fibrosis by ≥1 stage without worsening of NASH	NASH resolution, change in HFF by MRI-PDFF, non-invasive markers of fibrosis, glycaemic control, lipid metabolism, safety, tolerability, immunogenicity	Adults with NASH and fibrosis stages 2-3
**Kuchay, 2018**	RCT, open-label, controlled	NCT02686476	20 weeks	Control, Empagliflozin 10 mg daily	Change in liver fat content (MRI-PDFF)	Changes in AST, ALT, GGT levels	Adults with T2D and NAFLD

**Loomba, 2023_a**	RCT, DB, PC, phase 1b/2a	NCT04048135	12 weeks	Placebo, Pegozafermin 3 mg, 9 mg, 18 mg weekly, 27 mg weekly, 18 mg biweekly, 36 mg biweekly	Safety, tolerability, pharmacokinetics	Changes in hepatic fat fraction (MRI-PDFF), bodyweight, lipid profile, liver enzymes, immunogenicity	Adults with NASH
**Loomba, 2023_b**	Multinational, RCT, DB, PC, phase 2b	NCT04929483	24 weeks	Pegozafermin (15 mg, 30 mg weekly, 44 mg biweekly), Placebo	Improvement in liver fibrosis, NASH resolution	NAFLD activity score, liver variables, metabolic variables, safety	Patients with NASH, aged 21–75 years, fibrosis stage F2/F3, NAFLD score ≥4
**Loomba, 2023_c**	RCT, DB, PC phase 2 trial	NCT03987451	48 weeks	Semaglutide 2.4 mg once weekly vs placebo	Improvement in liver fibrosis without worsening NASH	Liver fat content change (MRI-PDFF), NASH resolution, fibrosis stage change, adverse events	Biopsy-confirmed NASH-related cirrhosis, BMI ≥27 kg/m^2
**Newsome, 2021**	RCT, DB, PC	NCT02970942	72 weeks	Semaglutide (0.1 mg, 0.2 mg, 0.4 mg daily), Placebo	Resolution of NASH without worsening of fibrosis	Changes in fibrosis stage, liver enzymes, metabolic parameters, safety	Patients with NASH, aged 18–75 years, fibrosis stage F1-F3, NAFLD score ≥4
**Sanyal, 2018**	RCT, DB, PC, phase 2a	NCT02413372	16 weeks	Placebo, Pegbelfermin 10 mg daily, 20 mg weekly	Safety, tolerability, hepatic fat fraction change	Pharmacokinetics, immunogenicity, exploratory endpoints	NASH patients
**Harrison, 2019**	DB, RCT, PC	NCT03987451	36 weeks	Resmetirom 80 mg, Placebo	Percent relative change in hepatic fat fraction by MRI-PDFF at 12 weeks	Proportions of patients with ≥30% hepatic fat reduction at 12 and 36 weeks; Absolute hepatic fat reduction at 12 and 36 weeks; Changes in liver enzymes, fibrosis biomarkers, and lipids	Adults with biopsy-confirmed NASH; ≥18 years; ≥10% hepatic fat on screening MRI-PDFF
**Harrison, 2023_a**	RCT, DB, PC, phase 3	NCT04197479	52 weeks	Resmetirom 100 mg OL, Resmetirom 100 mg DB, Resmetirom 80 mg DB, Placebo DB	Safety and tolerability of resmetirom in patients with NAFLD (presumed NASH)	Proportion of patients achieving ≥30% reduction in liver fat content (MRI-PDFF); Changes in liver volume, liver fat volume, VAT, SAT, body weight, waist circumference, BMI, liver enzymes, glucose metabolism, cardiovascular risk factors, and exploratory blood biomarkers	Adults ≥18 years with ≥3 metabolic risk factors; Patients with NAFLD (presumed NASH); Acceptable standard blood chemistry and hematology results; ≥8% hepatic fat (MRI-PDFF)
**Harrison, 2024**	RCT, DB, PC, phase 3	NCT03900429	52 weeks	Resmetirom 80 mg, Resmetirom 100 mg, Placebo	≥2 point reduction in NAFLD activity score without worsening fibrosis. Fibrosis improvement: ≥1 stage increase without worsening NAFLD activity score	Change in LDL cholesterol at week 24; Changes in liver enzymes and noninvasive tests	Adults with biopsy-confirmed NASH and fibrosis stages F1B, F2, or F3; 966 patients

Abbreviations:

- **RCT**: Randomized Controlled Trial
- **DB**: Double-Blind
- **PC**: Placebo-Controlled
- **NAFLD**: Non-Alcoholic Fatty Liver Disease
- **NASH**: Non-Alcoholic Steatohepatitis
- **MRI-PDFF**: Magnetic Resonance Imaging-Proton Density Fat Fraction
- **IHCL**: Intrahepatocellular Lipid
- **SAT**: Subcutaneous Adipose Tissue
- **VAT**: Visceral Adipose Tissue
- **MRE**: Magnetic Resonance Elastography
- **HFF**: Hepatic Fat Fraction
- **ALT**: Alanine Aminotransferase
- **AST**: Aspartate Aminotransferase
- **GGT**: Gamma-Glutamyl Transferase
- **BMI**: Body Mass Index
- **FPG**: Fasting Plasma Glucose
- **HbA1c**: Hemoglobin A1c

Supplement S3. Baseline Characteristics of the Participants

Table S3. Baseline Characteristics of Patients

The table summarizes the baseline characteristics of patients included in the study. Characteristics include the number of participants, age, male participants, body mass index (BMI), levels of alanine aminotransferase (ALT) and aspartate aminotransferase (AST), and glycated hemoglobin (HbA1c). Mean values with SD are provided for continuous variables, while percentages are provided for categorical variables.

Study	Participants	Age (years)	Sex (Male) (%)	BMI (kg/m^2)	ALT (U/L)	AST (U/L)	HbA1c (%)
**Abdelmalek,2024**	154	59.4(8.7)	36.0	35.6(6.1)	48.6(26.3)	45.5(24.1)	6.9(1.1)
**Armstrong,2016**	52	51.0(11.5)	60.0	35.9(5.5)	71.5(38.0)	51.0(24.5)	6.0(0.8)
**Flint,2021**	67	60.0(9.3)	65.0	35.4(5.9)	37.5(83.7)	30.0(67.2)	7.4(1.0)
**Guo,2020**	91	57.1(11.2)	46.0	34.6(7.5)	54.4(28.6)	29.5(16.3)	6.7(1.3)
**Harrison,2019**	125	50.2(11.5)	51.0	35.1(6.1)	52.6(30.8)	37.2(18.6)	6.3(1.1)
**Harrison,2021**	80	54.3(12.0)	48.0	37.7(6.8)	51.5(30.0)	37.4(17.4)	6.6(1.2)
**Harrison,2023_a**	1185	55.8(11.8)	51.8	35.5(6.1)	37.0(25.4)	25.7(14.1)	6.0(0.0)
**Harrison,2023_b**	30	51.1(11.6)	41.0	38.4(8.1)	58.6(29.2)	40.3(18.4)	6.3(1.0)
**Harrison,2023_c**	128	52.7(13.0)	38.1	37.5(7.3)	37.0(13.8)	37.0(13.8)	6.7(1.1)
**Harrison,2024**	966	56.7(11.0)	55.8	35.7(6.8)	54.6(32.0)	40.4(23.0)	-
**Kuchay_2018**	42	52.3(6.9)	-	29.7(3.5)	56.8(30.3)	44.9(23.9)	9.0(1.1)
**Loomba,2023_a**	81	51.9(9.8)	38.5	34.6(4.8)	55.4(39.2)	30.9(20.7)	9.0(1.1)
**Loomba,2023_b**	71	59.2(8.2)	30.0	35.0(5.9)	44.5(58.2)	44.4(45.8)	7.2(1.3)
**Loomba,2023_c**	71	55.5(10.5)	34.0	36.8(5.6)	56.8(30.6)	44.0(23.0)	6.8(1.2)
**Newsome,2021**	320	55.0(10.5)	58.0	35.8(6.4)	54.0(86.0)	43.0(79.0)	7.3(1.2)
**Sanyal,2018**	75	50.3(11.6)	35.8	35.4(5.6)	42.5(22.4)	53.5(33.4)	6.1(1.0)

Abbreviations:

- **NASH:** Non-alcoholic steatohepatitis
- **BMI:** Body mass index
- **ALT:** Alanine aminotransferase
- **AST:** Aspartate aminotransferase
- **HbA1c:** Glycated hemoglobin

Supplement S4. Risk of bias assessment of included trials for each outcome

Table S4.1 NASH resolution

Study	Randomization process	Deviations from intended interventions	Missing outcome data	Measurement of the outcome	Selection of the reported result	Overall
**Abdelmalek, 2024**	low	low	low	low	some concern	some concern
**Armstrong, 2016**	low	low	low	low	low	low
**Flint, 2021**	low	low	low	low	low	low
**Guo, 2020**	low	some concern	low	high	low	some concern
**Harrison, 2019**	low	low	low	low	low	low
**Harrison, 2021**	low	low	low	some concern	high	some concern
**Harrison, 2023 a**	low	some concern	high	some concern	low	some concern
**Harrison, 2023 b**	low	low	some concern	low	low	some concern
**Harrison, 2023 c**	low	low	some concern	low	low	some concern
**Harrison, 2024**	low	low	low	low	low	low
**Kuchay, 2018**	low	high	low	low	low	low
**Loomba, 2023, c**	low	low	low	low	low	low
**Loomba, 2023 a**	low	low	low	low	low	low
**Loomba, 2023 b**	low	low	low	low	low	low
**Newsome, 2021**	low	low	low	some concern	low	some concern
**Sanyal, 2018**	low	low	low	low	low	low

Table S4.2 Improvement in Fibrosis

Study	Randomization process	Deviations from intended interventions	Missing outcome data	Measurement of the outcome	Selection of the reported result	Overall
**Abdelmalek, 2024**	low	low	low	low	some concern	some concern
**Armstrong, 2016**	low	low	low	low	low	low
**Harrison, 2021**	low	low	low	some concern	high	some concern
**Harrison, 2023 a**	low	some concern	high	some concern	low	some concern
**Harrison, 2023 b**	low	low	some concern	low	low	some concern
**Harrison, 2023 c**	low	low	some concern	low	low	some concern
**Harrison, 2024**	low	low	low	low	low	low
**Loomba, 2023 c**	low	low	low	low	low	low
**Loomba, 2023 b**	low	low	low	low	low	low
**Newsome, 2021**	low	low	low	some concern	low	some concern

Table S4.3 Hepatic fat reduction (MRI PDFF)

Study	Randomization process	Deviations from intended interventions	Missing outcome data	Measurement of the outcome	Selection of the reported result	Overall
**Flint, 2021**	low	low	low	low	low	low
**Guo, 2020**	low	some concern	low	high	low	some concern
**Harrison, 2019**	low	low	low	low	low	low
**Harrison, 2021**	low	low	low	some concern	high	some concern
**Harrison, 2023 a**	low	some concern	high	some concern	low	some concern
**Harrison, 2024**	low	low	low	low	low	low
**Kuchay, 2018**	low	high	low	low	low	low
**Loomba, 2023, c**	low	low	low	low	low	low
**Loomba, 2023 a**	low	low	low	low	low	low
**Loomba, 2023 b**	low	low	low	low	low	low
**Sanyal, 2018**	low	low	low	low	low	low

Table S4.4 >30% Fat Reduction on MRI-PDFF

Study	Randomization process	Deviations from intended interventions	Missing outcome data	Measurement of the outcome	Selection of the reported result	Overall
**Abdelmalek, 2024**	low	low	low	low	some concern	some concern
**Armstrong, 2016**	low	low	low	low	low	low
**Flint, 2021**	low	low	low	low	low	low
**Harrison, 2019**	low	low	low	low	low	low
**Harrison, 2021**	low	low	low	some concern	high	some concern
**Harrison, 2023 b**	low	low	some concern	low	low	some concern
**Harrison, 2024**	low	low	low	low	low	low
**Loomba, 2023 a**	low	low	low	low	low	low
**Loomba, 2023 b**	low	low	low	low	low	low
**Newsome, 2021**	low	low	low	some concern	low	some concern
**Sanyal, 2018**	low	low	low	low	low	low

Table S4.5 Change in VCTE

Study	Randomization process	Deviations from intended interventions	Missing outcome data	Measurement of the outcome	Selection of the reported result	Overall
**Flint, 2021**	low	low	low	low	low	low
**Harrison, 2023 a**	low	some concern	high	some concern	low	some concern
**Harrison, 2023 b**	low	low	some concern	low	low	some concern
**Harrison, 2023 c**	low	low	some concern	low	low	some concern
**Harrison, 2024**	low	low	low	low	low	low
**Kuchay, 2018**	low	high	low	low	low	low
**Loomba, 2023 c**	low	low	low	low	low	low
**Loomba, 2023 b**	low	low	low	low	low	low
**Newsome, 2021**	low	low	low	some concern	low	some concern
**Sanyal, 2018**	low	low	low	low	low	low

Table S4.6 Change in ALT

Study	Randomization process	Deviations from intended interventions	Missing outcome data	Measurement of the outcome	Selection of the reported result	Overall
**Abdelmalek, 2024**	low	low	low	low	some concern	some concern
**Armstrong, 2016**	low	low	low	low	low	low
**Flint, 2021**	low	low	low	low	low	low
**Guo, 2020**	low	some concern	low	high	low	some concern
**Harrison, 2023 a**	low	some concern	high	some concern	low	some concern
**Harrison, 2023 c**	low	low	some concern	low	low	some concern
**Harrison, 2024**	low	low	low	low	low	low
**Kuchay, 2018**	low	high	low	low	low	low
**Loomba, 2023 c**	low	low	low	low	low	low
**Loomba, 2023 a**	low	low	low	low	low	low
**Loomba, 2023 b**	low	low	low	low	low	low
**Newsome, 2021**	low	low	low	some concern	low	some concern
**Sanyal, 2018**	low	low	low	low	low	low

Table S4.7 Change in AST

Study	Randomization process	Deviations from intended interventions	Missing outcome data	Measurement of the outcome	Selection of the reported result	Overall
**Abdelmalek, 2024**	low	low	low	low	some concern	some concern
**Armstrong, 2016**	low	low	low	low	low	low
**Flint, 2021**	low	low	low	low	low	low
**Guo, 2020**	low	some concern	low	high	low	some concern
**Harrison, 2023 a**	low	some concern	high	some concern	low	some concern
**Harrison, 2023 c**	low	low	some concern	low	low	some concern
**Harrison, 2024**	low	low	low	low	low	low
**Kuchay, 2018**	low	high	low	low	low	low
**Loomba, 2023 c**	low	low	low	low	low	low
**Loomba, 2023 a**	low	low	low	low	low	low
**Loomba, 2023 b**	low	low	low	low	low	low
**Newsome, 2021**	low	low	low	some concern	low	some concern
**Sanyal, 2018**	low	low	low	low	low	low

Table S4.8 Change in GGT

Study	Randomization process	Deviations from intended interventions	Missing outcome data	Measurement of the outcome	Selection of the reported result	Overall
**Armstrong, 2016**	low	low	low	low	low	low
**Flint, 2021**	low	low	low	low	low	low
**Guo, 2020**	low	some concern	low	high	low	some concern
**Harrison, 2023 a**	low	some concern	high	some concern	low	some concern
**Harrison, 2023 c**	low	low	some concern	low	low	some concern
**Harrison, 2024**	low	low	low	low	low	low
**Kuchay, 2018**	low	high	low	low	low	low
**Loomba, 2023, c**	low	low	low	low	low	low
**Loomba, 2023 b**	low	low	low	low	low	low
**Newsome, 2021**	low	low	low	some concern	low	some concern

Table S4.9 Adverse events

Study	Randomization process	Deviations from intended interventions	Missing outcome data	Measurement of the outcome	Selection of the reported result	Overall
**Abdelmalek, 2024**	low	low	low	low	some concern	some concern
**Armstrong, 2016**	low	low	low	low	low	low
**Flint, 2021**	low	low	low	low	low	low
**Harrison, 2023 a**	low	some concern	high	some concern	low	some concern
**Harrison, 2023 b**	low	low	some concern	low	low	some concern
**Harrison, 2023 c**	low	low	some concern	low	low	some concern
**Harrison, 2024**	low	low	low	low	low	low
**Kuchay, 2018**	low	high	low	low	low	low
**Loomba, 2023, c**	low	low	low	low	low	low
**Loomba, 2023 a**	low	low	low	low	low	low
**Loomba, 2023 b**	low	low	low	low	low	low
**Newsome, 2021**	low	low	low	some concern	low	some concern
**Sanyal, 2018**	low	low	low	low	low	low

Table S4.10 Treatment discontinuation

Study	Randomization process	Deviations from intended interventions	Missing outcome data	Measurement of the outcome	Selection of the reported result	Overall
**Abdelmalek, 2024**	low	low	low	low	some concern	some concern
**Armstrong, 2016**	low	low	low	low	low	low
**Flint, 2021**	low	low	low	low	low	low
**Harrison, 2023 a**	low	some concern	high	some concern	low	some concern
**Harrison, 2023 b**	low	low	some concern	low	low	some concern
**Harrison, 2023 c**	low	low	some concern	low	low	some concern
**Harrison, 2024**	low	low	low	low	low	low
**Kuchay, 2018**	low	high	low	low	low	low
**Loomba, 2023, c**	low	low	low	low	low	low
**Loomba, 2023 a**	low	low	low	low	low	low
**Loomba, 2023 b**	low	low	low	low	low	low
**Newsome, 2021**	low	low	low	some concern	low	some concern
**Sanyal, 2018**	low	low	low	low	low	low

Table S4.11 Nausea

Study	Randomization process	Deviations from intended interventions	Missing outcome data	Measurement of the outcome	Selection of the reported result	Overall
**Abdelmalek, 2024**	low	low	low	low	some concern	some concern
**Armstrong, 2016**	low	low	low	low	low	low
**Flint, 2021**	low	low	low	low	low	low
**Guo, 2020**	low	some concern	low	high	low	some concern
**Harrison, 2019**	low	low	low	low	low	low
**Harrison, 2021**	low	low	low	some concern	high	some concern
**Harrison, 2023 a**	low	some concern	high	some concern	low	some concern
**Harrison, 2023 b**	low	low	some concern	low	low	some concern
**Harrison, 2023 c**	low	low	some concern	low	low	some concern
**Harrison, 2024**	low	low	low	low	low	low
**Kuchay, 2018**	low	high	low	low	low	low
**Loomba, 2023, c**	low	low	low	low	low	low
**Loomba, 2023 a**	low	low	low	low	low	low
**Loomba, 2023 b**	low	low	low	low	low	low
**Newsome, 2021**	low	low	low	some concern	low	some concern
**Sanyal, 2018**	low	low	low	low	low	low

Table S4.12 Diarrhea

Study	Randomization process	Deviations from intended interventions	Missing outcome data	Measurement of the outcome	Selection of the reported result	Overall
**Abdelmalek, 2024**	low	low	low	low	some concern	some concern
**Armstrong, 2016**	low	low	low	low	low	low
**Flint, 2021**	low	low	low	low	low	low
**Harrison, 2023 a**	low	some concern	high	some concern	low	some concern
**Harrison, 2023 b**	low	low	some concern	low	low	some concern
**Harrison, 2023 c**	low	low	some concern	low	low	some concern
**Harrison, 2024**	low	low	low	low	low	low
**Kuchay, 2018**	low	high	low	low	low	low
**Loomba, 2023, c**	low	low	low	low	low	low
**Loomba, 2023 a**	low	low	low	low	low	low
**Loomba, 2023 b**	low	low	low	low	low	low
**Newsome, 2021**	low	low	low	some concern	low	some concern
**Sanyal, 2018**	low	low	low	low	low	low

Supplement S5. Network plots of treatment comparisons

Figure S5.1 Network Plot of Treatment Comparisons for NASH Resolution

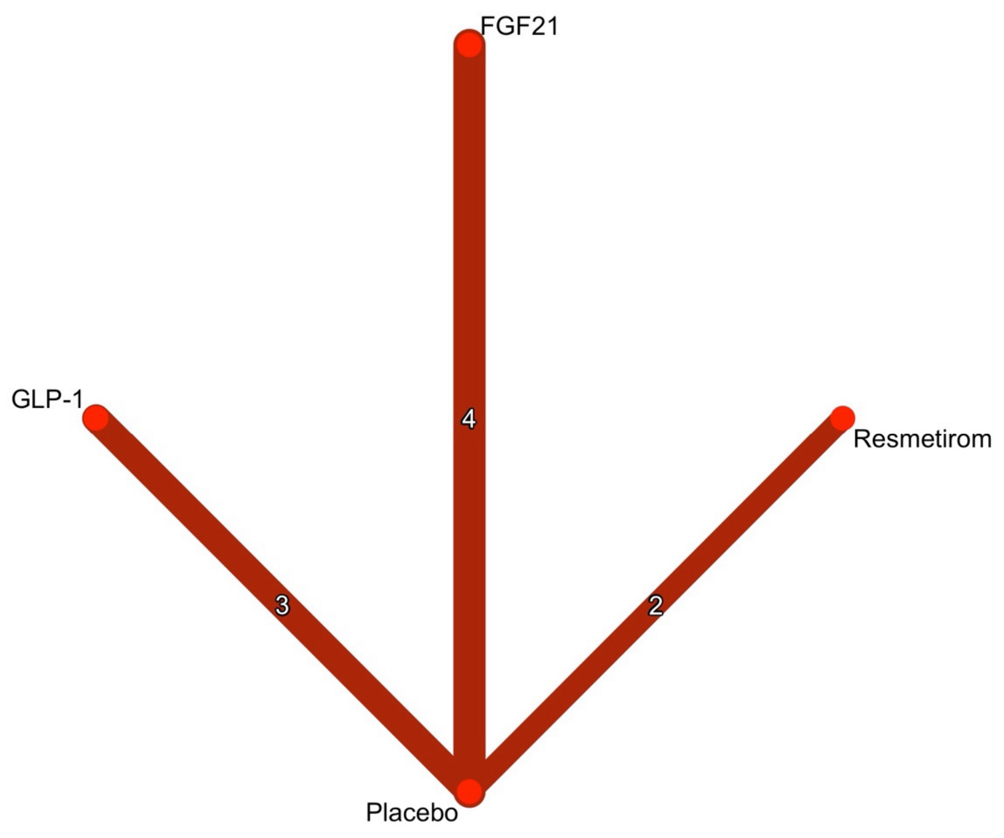

Figure S5.2 Network Plot of Treatment Comparisons for improvement in fibrosis

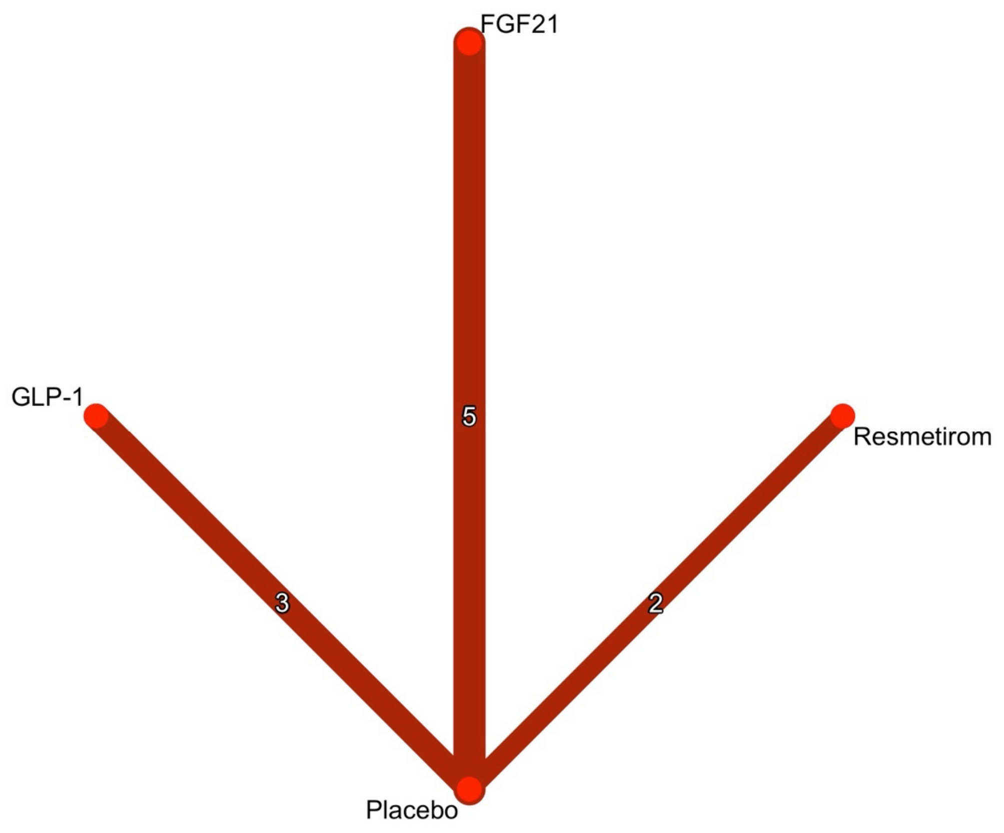

Figure S5.3 Network Plot of Treatment Comparisons for change in MRI-PDFF

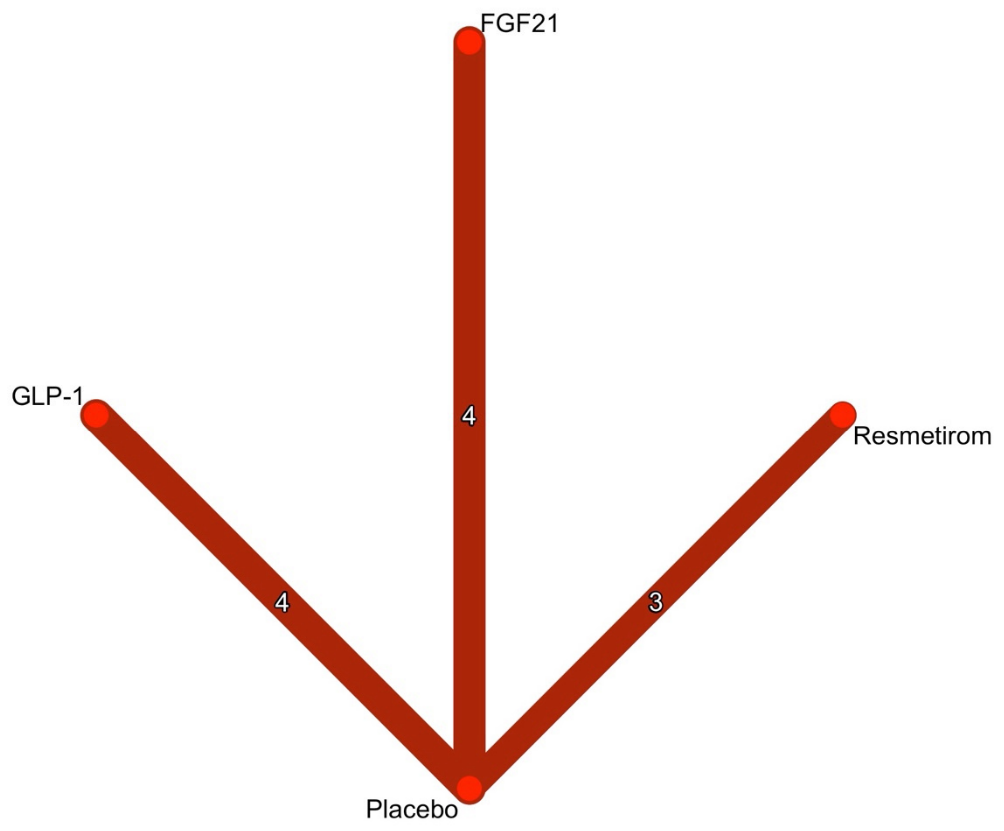

Figure S5.4 Network Plot of Treatment Comparisons for >30% Fat Reduction on MRI-PDFF

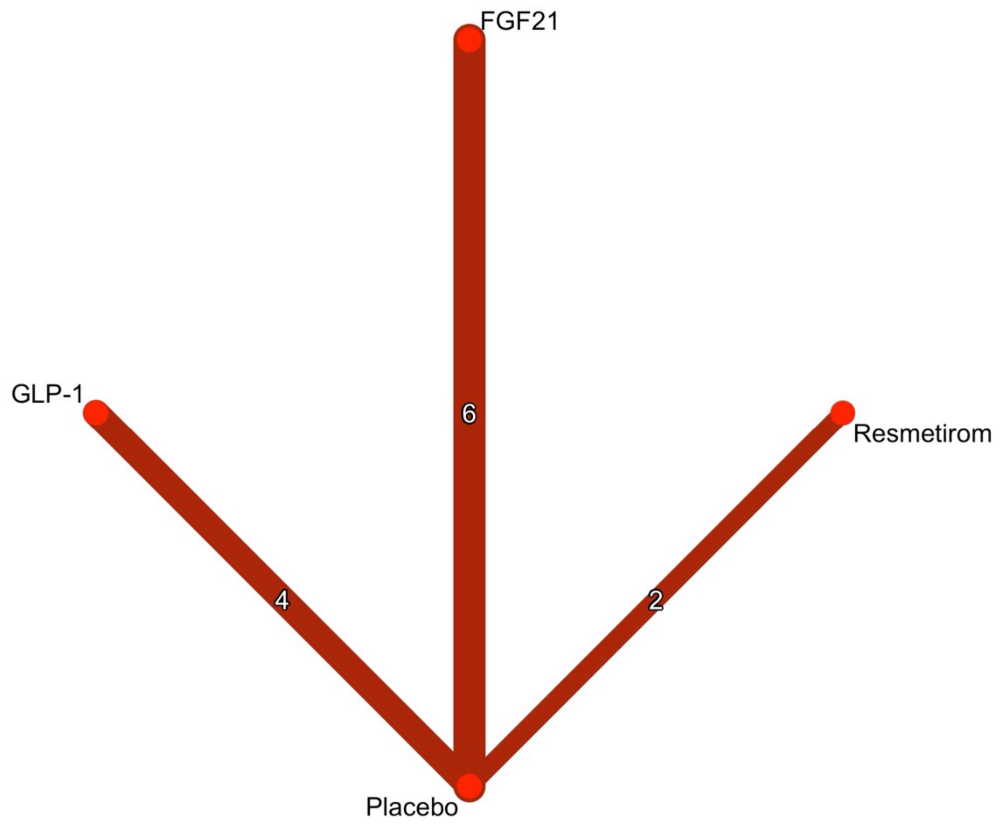

Figure S5.5 Network Plot of Treatment Comparisons for Change in VCTE

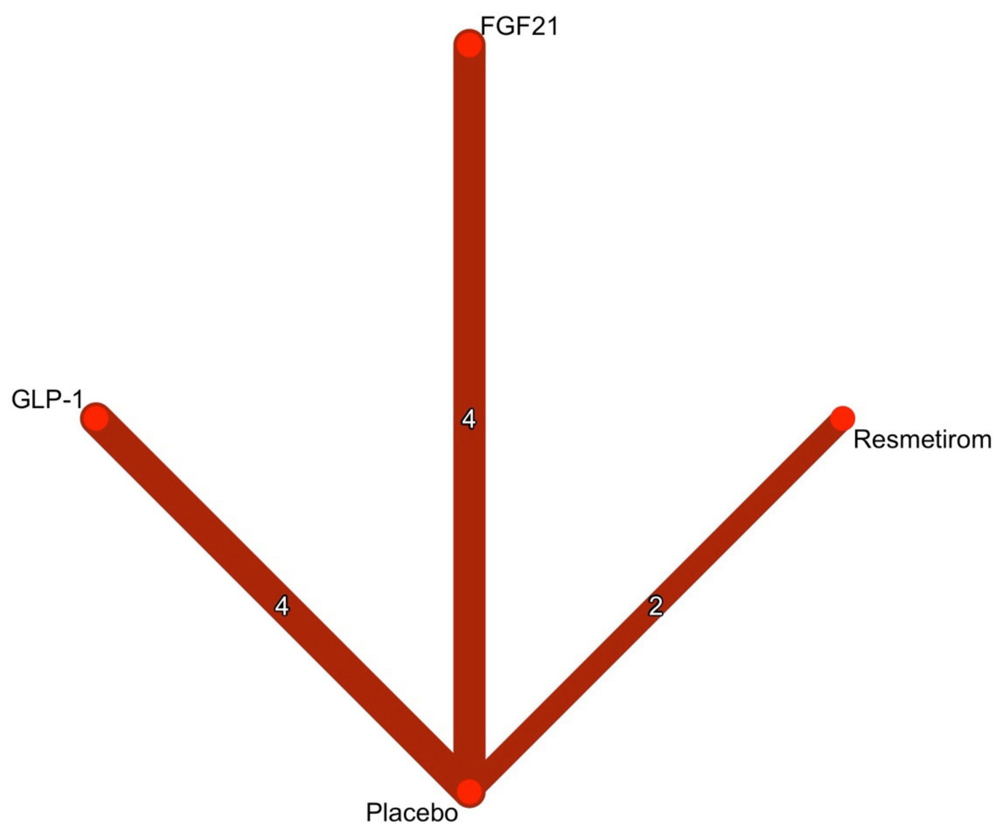

Figure S5.6. Network Plot of Treatment Comparisons for change in ALT

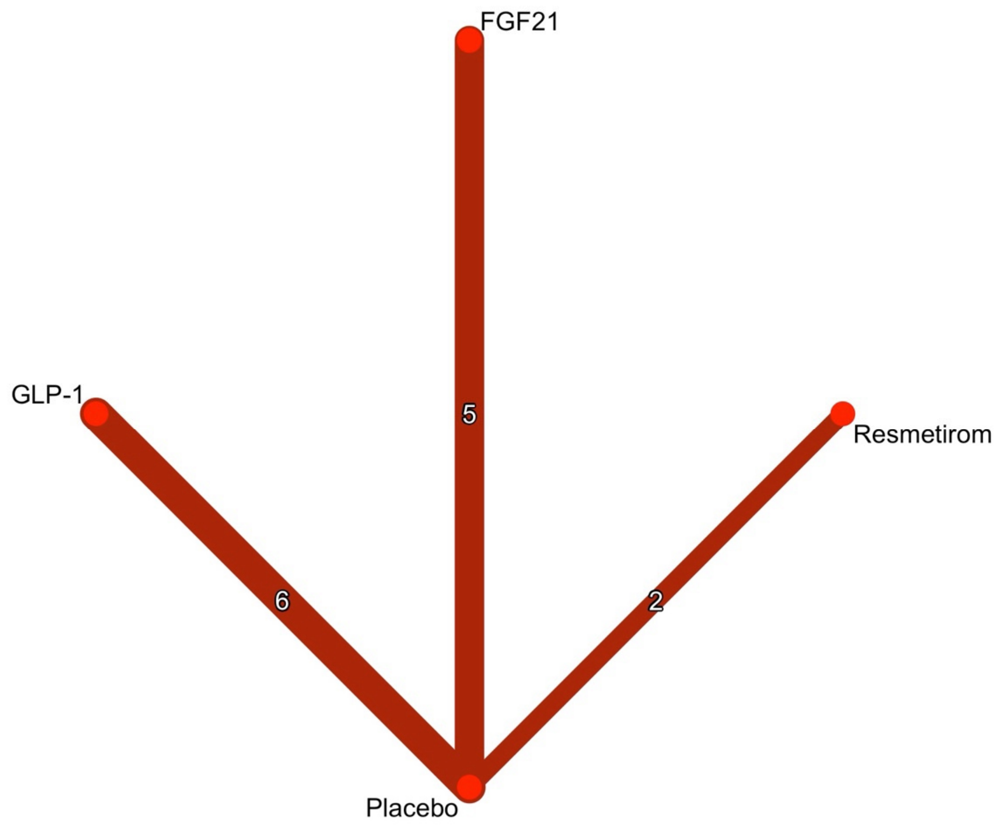

Figure S5.7 Network Plot of Treatment Comparisons for change in AST

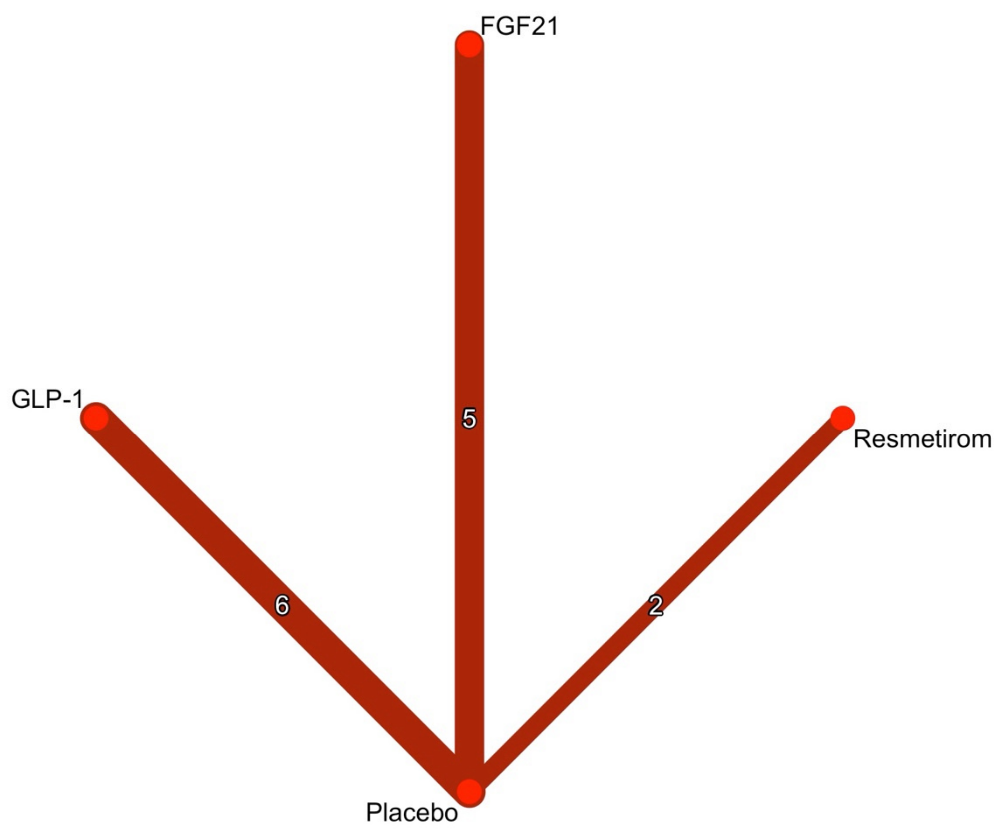

Figure S5.8 Network Plot of Treatment Comparisons for change in GGT

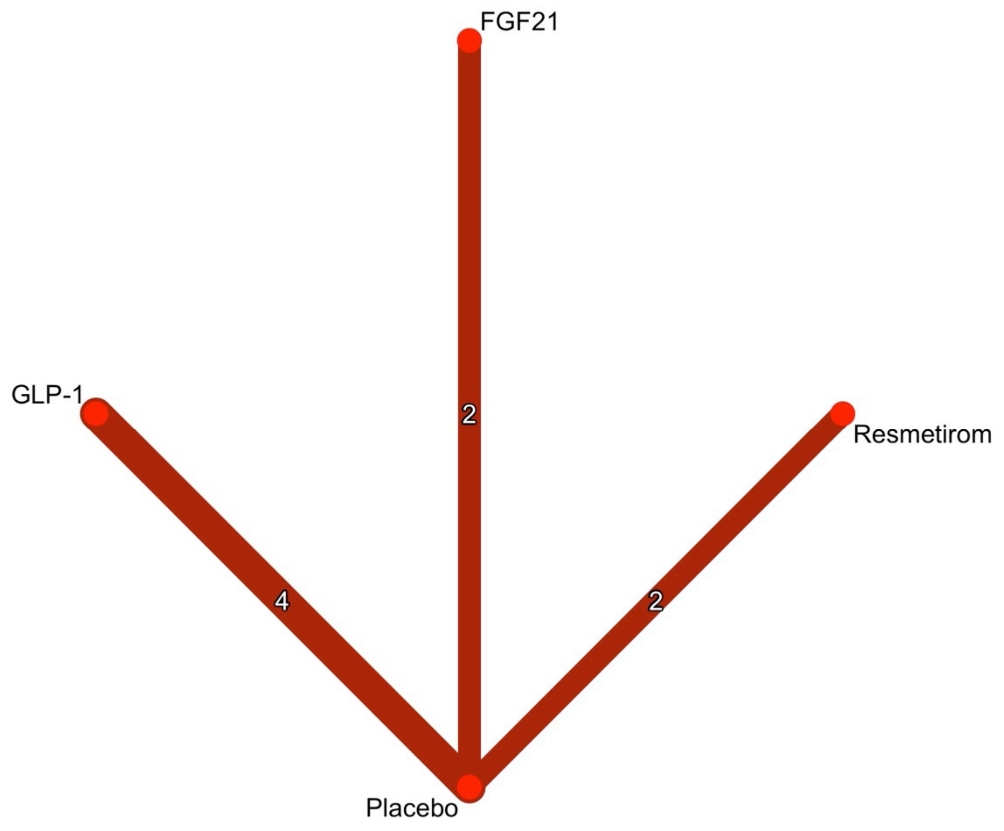

Figure S5.9 Network Plot of Treatment Comparisons for adverse events

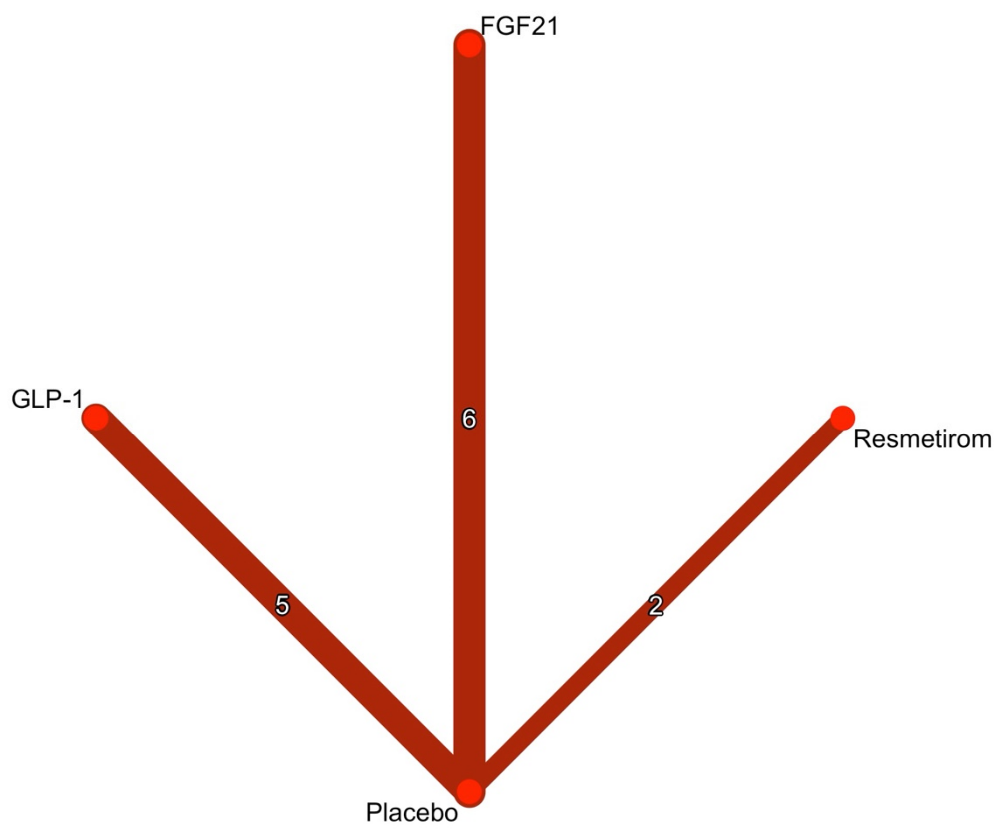

Figure S5.10 Network Plot of Treatment Comparisons for treatment discontinuation

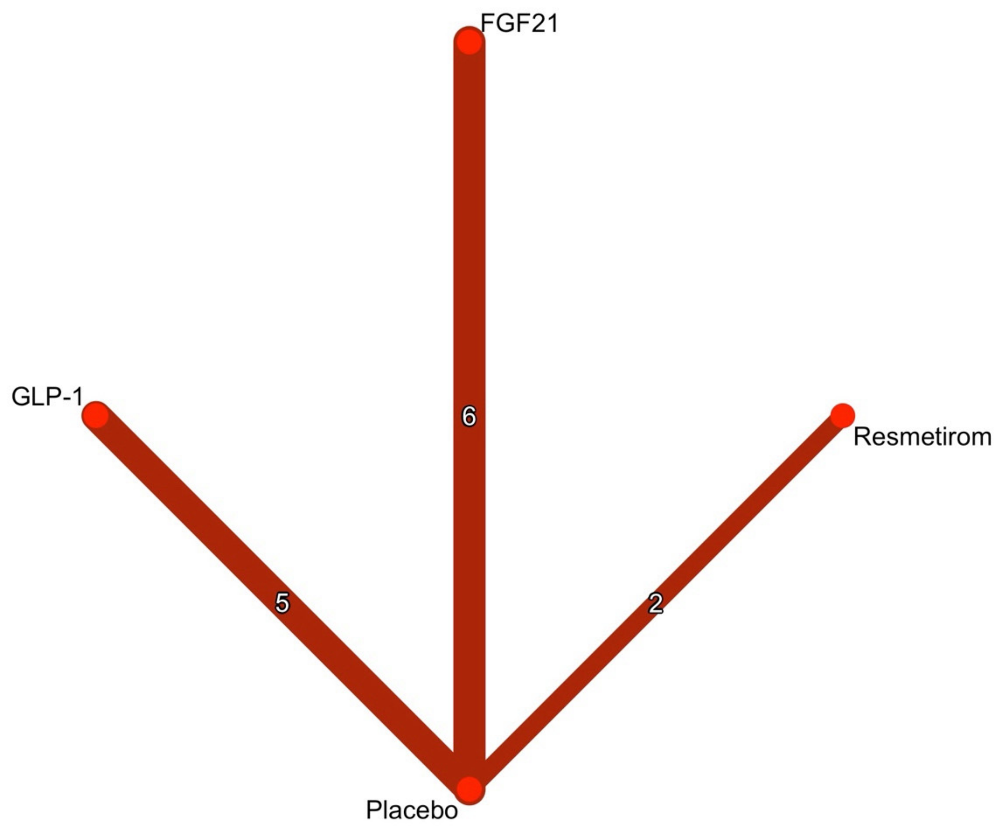

Figure S5.11 Network Plot of Treatment Comparisons for nausea

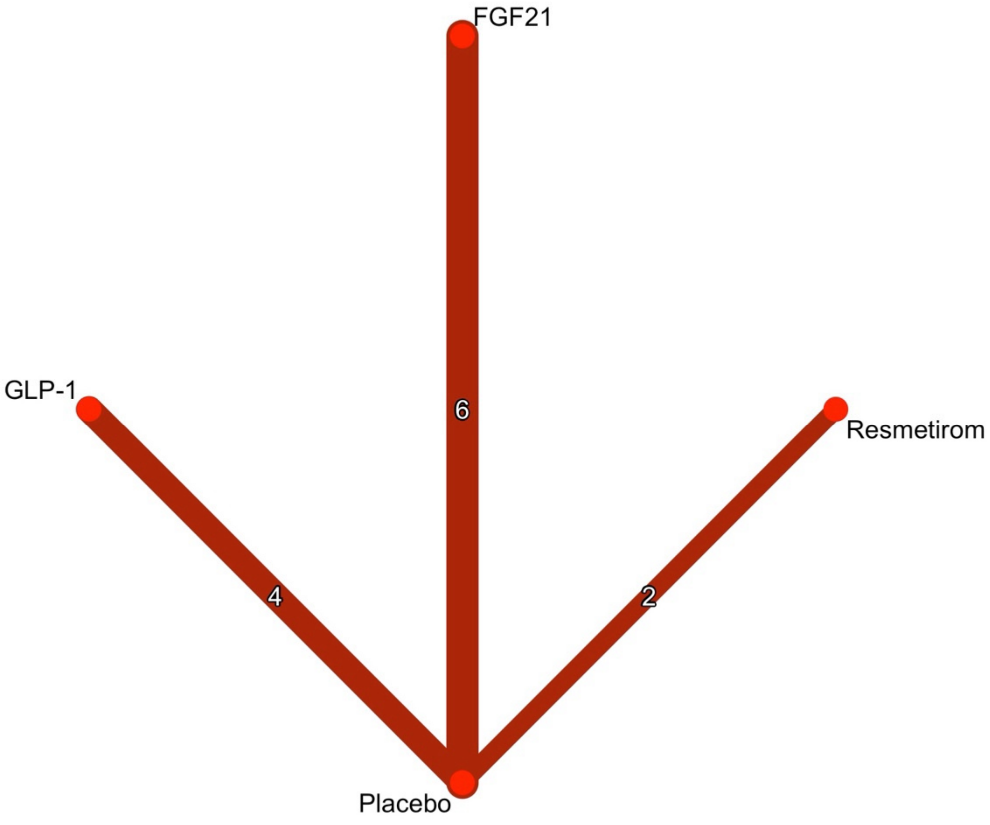

Figure S5.12**.** Network Plot of Treatment Comparisons for diarrhea

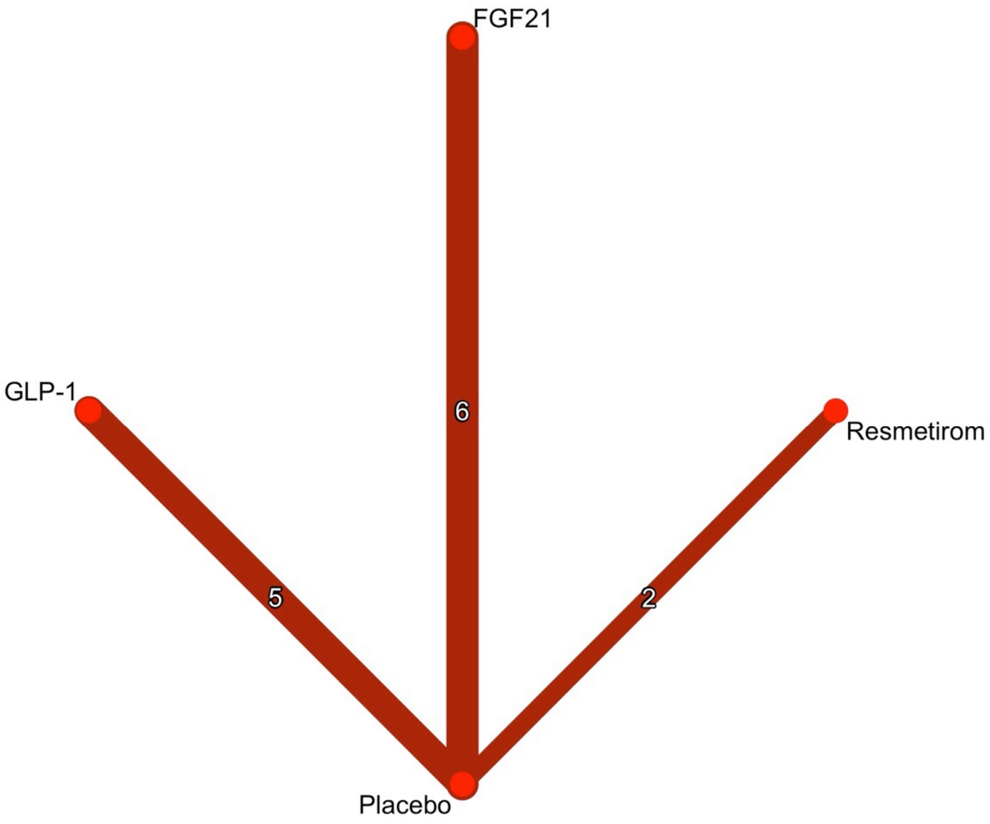

Supplement S6. Publication bias (funnel plot)

Figure S6.1 NASH resolution

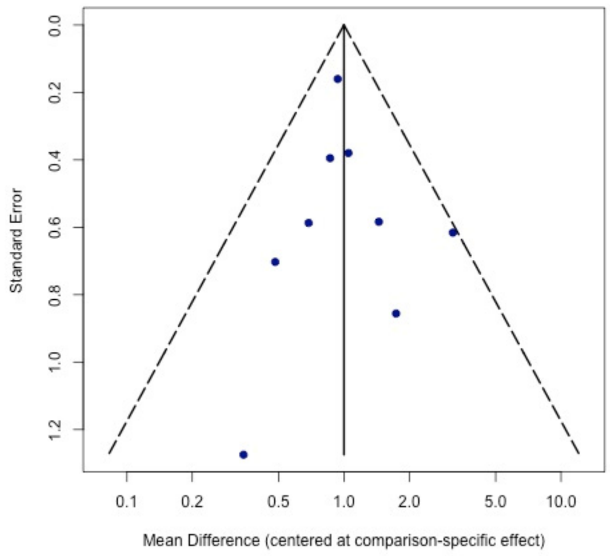

Figure S6.2 Improvement in Fibrosis

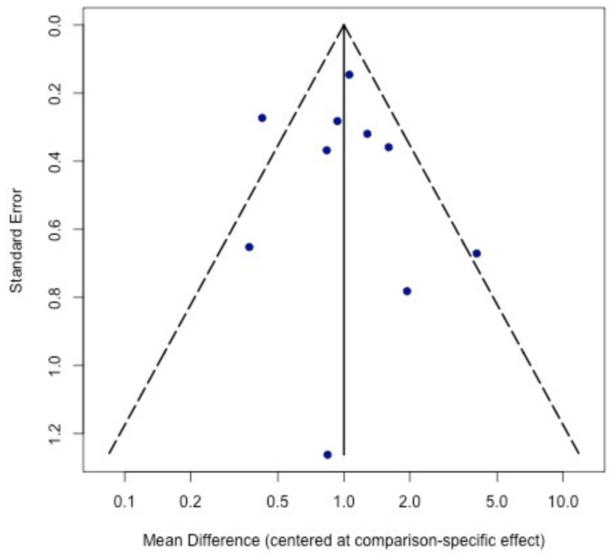

Figure S6.3 Hepatic fat reduction (MRI PDFF)

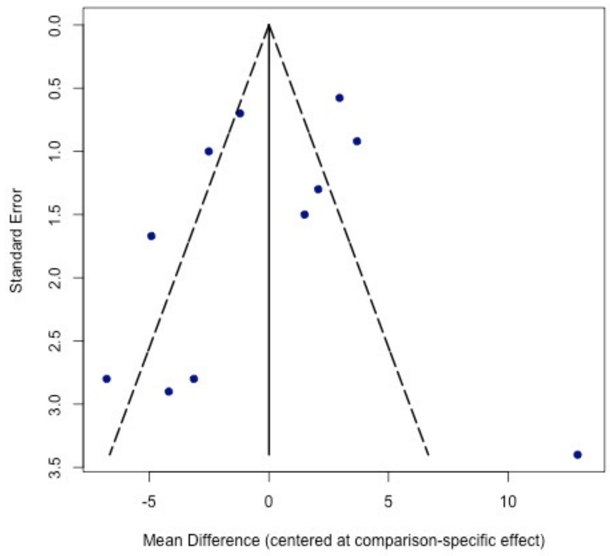

Figure S6.4 >30% Fat Reduction on MRI-PDFF

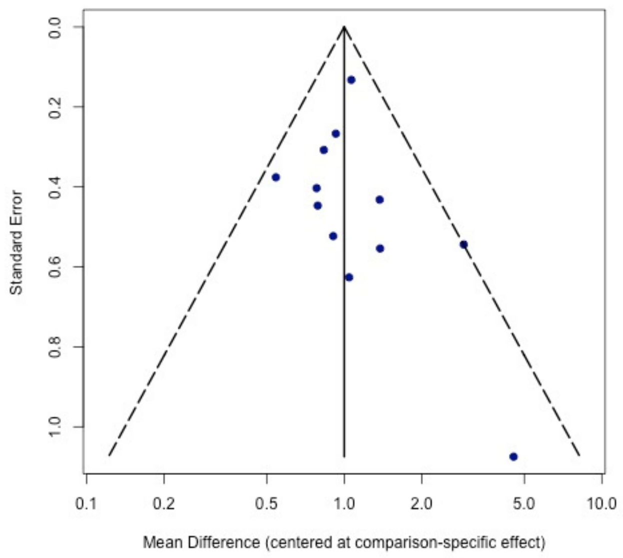

Figure S6.5 Change in VCTE

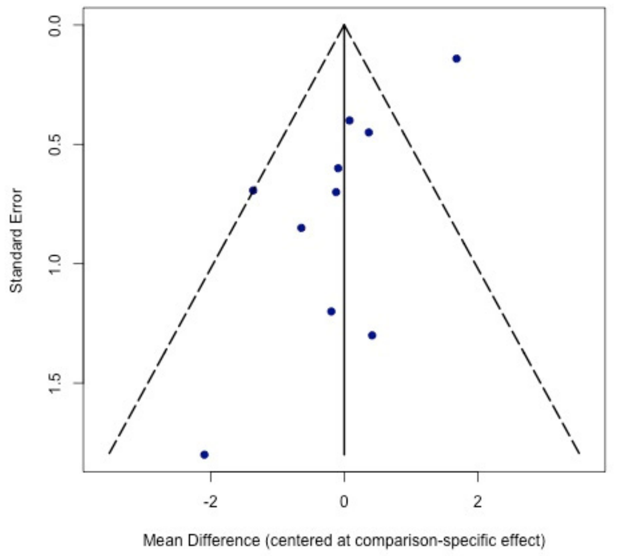

Figure S6.6 Change in ALT

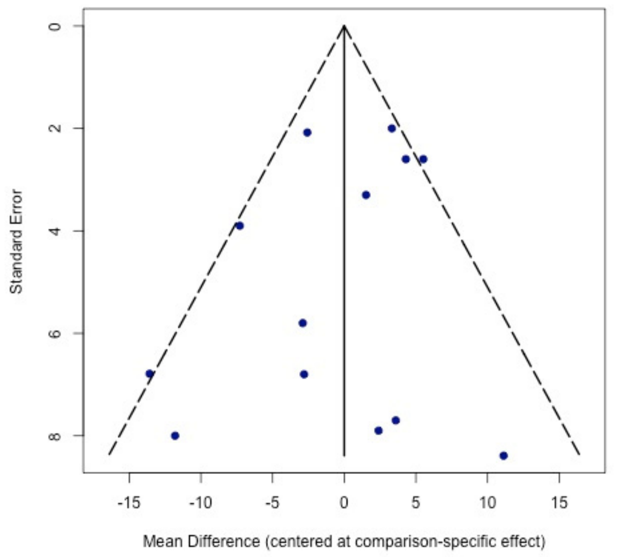

Figure S6.7 Change in AST

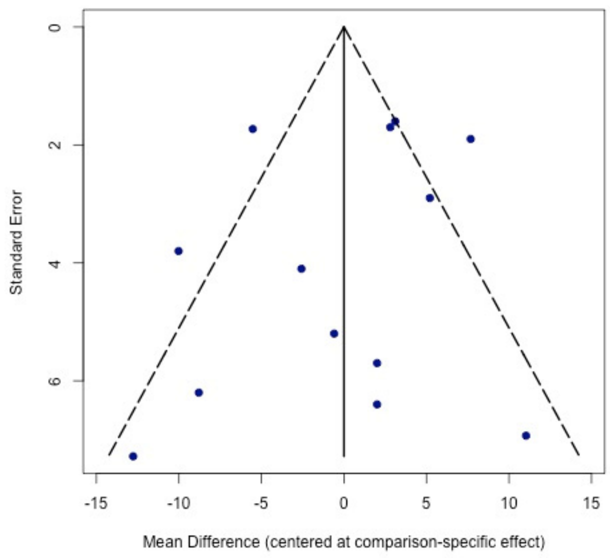

Figure S6.8 Change in GGT

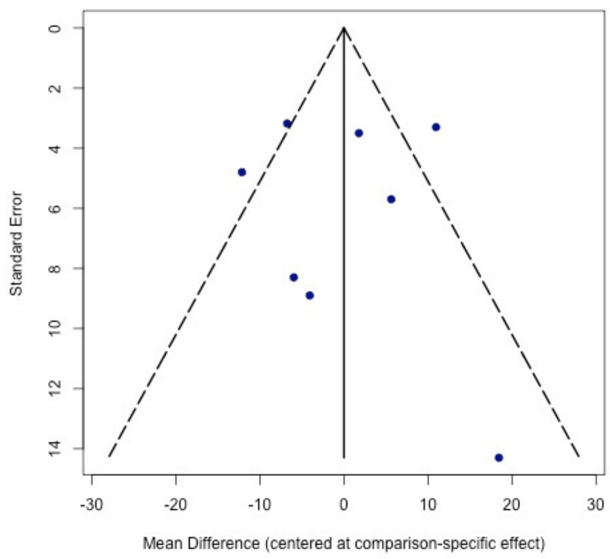

Figure S6.9 Adverse events

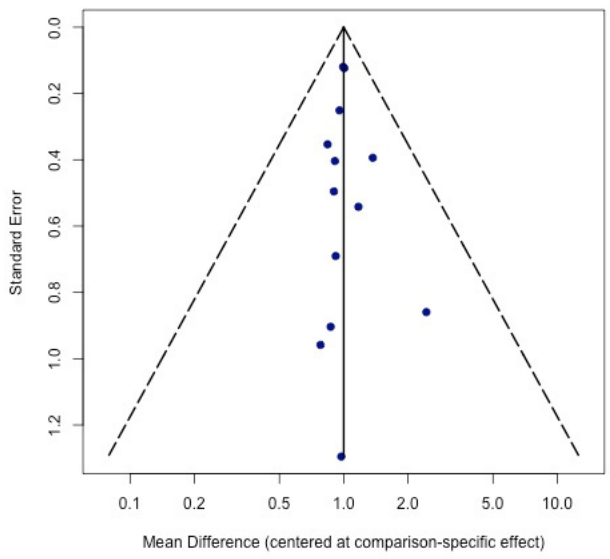

Figure S6.10 Treatment discontinuation

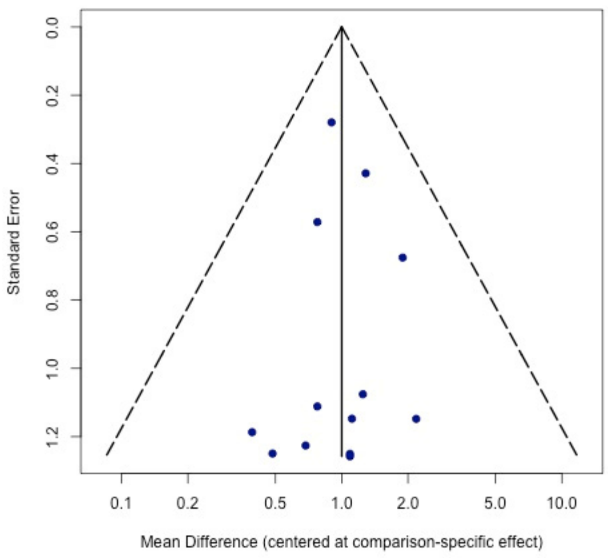

Figure S6.11 Nausea

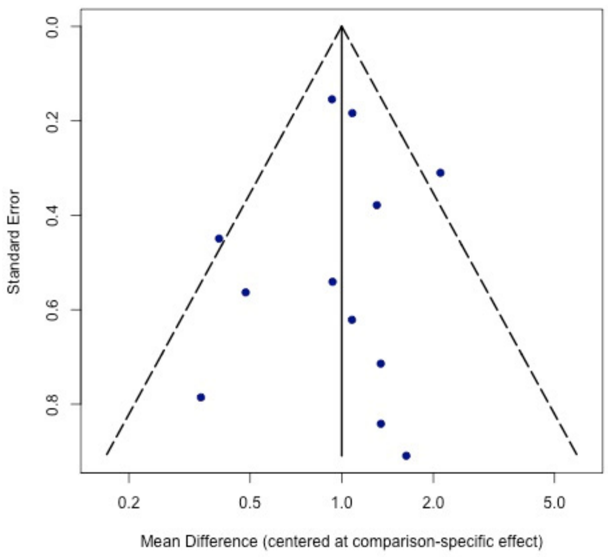

Figure S6.12 Diarrhea

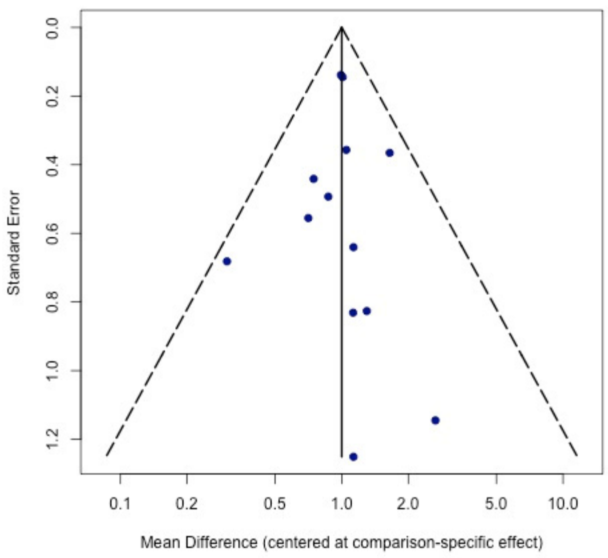

Supplement S7. Certainty of the effect estimates

Table S7.1 NASH resolution

FGF21	.	.	4.83 (2.72; 8.56)
1.63 (0.85; 3.12)	Resmetirom	.	2.96 (2.18; 4.00)
1.95 (0.86; 4.43)	1.19 (0.62; 2.31)	GLP-1	2.48 (1.38; 4.45)
4.83 (2.72; 8.56)	2.96 (2.18; 4.00)	2.48 (1.38; 4.45)	Placebo

Table S7.2 Improvement in Fibrosis

FGF21	.	.	2.03 (1.43; 2.88)
1.18 (0.77; 1.82)	Resmetirom	.	1.72 (1.33; 2.21)
1.89 (1.00; 3.57)	1.60 (0.89; 2.88)	GLP-1	1.07 (0.63; 1.82)
2.03 (1.43; 2.88)	1.72 (1.33; 2.21)	1.07 (0.63; 1.82)	Placebo

Table S7.3 Hepatic fat reduction (MRI PDFF)

Placebo	5.30 (4.20; 6.39)	6.77 (5.94; 7.60)	19.15 (15.76; 22.55)
5.30 (4.20; 6.39)	GLP-1	.	.
6.77 (5.94; 7.60)	1.47 (0.10; 2.85)	FGF21	.
19.15 (15.76; 22.55)	13.85 (10.29; 17.42)	12.38 (8.89; 15.88)	Resmetirom

Table S7.4 >30% Fat Reduction on MRI-PDFF

Resmetirom	.	.	3.68 (2.88; 4.71)
1.29 (0.84; 1.97)	FGF21	.	2.86 (2.03; 4.04)
2.03 (1.27; 3.26)	1.58 (0.93; 2.69)	GLP-1	1.81 (1.21; 2.71)
3.68 (2.88; 4.71)	2.86 (2.03; 4.04)	1.81 (1.21; 2.71)	Placebo

Table S7.5 Change in VCTE

Placebo	0.19 (-0.08; 0.46)	0.56 (-0.09; 1.22)	1.65 (1.00; 2.30)
0.19 (-0.08; 0.46)	FGF21	.	.
0.56 (-0.09; 1.22)	0.37 (-0.33; 1.08)	GLP-1	.
1.65 (1.00; 2.30)	1.46 (0.76; 2.17)	1.09 (0.16; 2.01)	Resmetirom

Table S7.6 Change in ALT

Placebo	8.67 (4.73; 12.61)	12.93 (10.42; 15.44)	14.14 (9.90; 18.38)
8.67 (4.73; 12.61)	GLP-1	.	.
12.93 (10.42; 15.44)	4.26 (-0.41; 8.93)	FGF21	.
14.14 (9.90; 18.38)	5.47 (-0.32; 11.26)	1.21 (-3.72; 6.14)	Resmetirom

Table S7.7 Change in AST

Placebo	7.61 (5.75; 9.47)	7.61 (4.01; 11.21)	8.14 (4.81; 11.47)
7.61 (5.75; 9.47)	FGF21	.	.
7.61 (4.01; 11.21)	-0.00 (-4.06; 4.05)	GLP-1	.
8.14 (4.81; 11.47)	0.53 (-3.29; 4.34)	0.53 (-4.37; 5.44)	Resmetirom

Table S7.8 Change in GGT

Placebo	16.01 (10.68; 21.34)	17.01 (10.93; 23.10)	17.44 (12.19; 22.68)
16.01 (10.68; 21.34)	Resmetirom	.	.
17.01 (10.93; 23.10)	1.00 (-7.09; 9.09)	FGF21	.
17.44 (12.19; 22.68)	1.42 (-6.06; 8.90)	0.42 (-7.61; 8.46)	GLP-1

Table S7.9 Adverse events

Placebo	0.82 (0.60; 1.13)	0.81 (0.46; 1.43)	0.68 (0.58; 0.81)
0.82 (0.60; 1.13)	FGF21	.	.
0.81 (0.46; 1.43)	0.99 (0.52; 1.90)	GLP-1	.
0.68 (0.58; 0.81)	0.83 (0.58; 1.19)	0.84 (0.46; 1.52)	Resmetirom

Table S7.10 Treatment discontinuation

Placebo	0.58 (0.37; 0.92)	0.54 (0.24; 1.22)	0.46 (0.20; 1.03)
0.58 (0.37; 0.92)	Resmetirom	.	.
0.54 (0.24; 1.22)	0.93 (0.37; 2.36)	GLP-1	.
0.46 (0.20; 1.03)	0.78 (0.31; 1.98)	0.84 (0.27; 2.63)	FGF21

Table S7.11 Nausea

Placebo	0.66 (0.44; 0.99)	0.57 (0.45; 0.72)	0.39 (0.24; 0.66)
0.66 (0.44; 0.99)	FGF21	.	.
0.57 (0.45; 0.72)	0.87 (0.54; 1.39)	Resmetirom	.
0.39 (0.24; 0.66)	0.60 (0.31; 1.15)	0.69 (0.39; 1.21)	GLP-1

Table S7.12 Diarrhea

Placebo	0.56 (0.34; 0.93)	0.53 (0.35; 0.81)	0.51 (0.42; 0.62)
0.56 (0.34; 0.93)	GLP-1	.	.
0.53 (0.35; 0.81)	0.94 (0.49; 1.81)	FGF21	.
0.51 (0.42; 0.62)	0.90 (0.53; 1.54)	0.96 (0.60; 1.54)	Resmetirom

Supplement S8. Certainty of the effect estimates

Table S8.1 NASH resolution

Comparison	Number of studies	Within-study bias	Reporting bias	Indirectness	Imprecision	Heterogeneity	Incoherence	Confidence rating	Reason(s) for downgrading
FGF21:Placebo	4	Some concerns	Low risk	No concerns	No concerns	No concerns	Major concerns	Low	["Within-study bias","Incoherence"]
GLP-1:Placebo	3	Some concerns	Low risk	No concerns	No concerns	Major concerns	Major concerns	Low	["Within-study bias","Heterogeneity","Incoherence"]
Placebo:Resmetirom	2	No concerns	Low risk	No concerns	No concerns	No concerns	Major concerns	Low	["Incoherence"]
FGF21:GLP-1	0	Some concerns	Low risk	No concerns	Major concerns	No concerns	Major concerns	Low	["Within-study bias","Imprecision","Incoherence"]
FGF21:Resmetirom	0	No concerns	Low risk	No concerns	Major concerns	No concerns	Major concerns	Low	["Imprecision","Incoherence"]
GLP-1:Resmetirom	0	No concerns	Low risk	No concerns	Major concerns	No concerns	Major concerns	Low	["Imprecision","Incoherence"]

Table S8.2 Improvement in Fibrosis

Comparison	Number of studies	Within-study bias	Reporting bias	Indirectness	Imprecision	Heterogeneity	Incoherence	Confidence rating	Reason(s) for downgrading
FGF21:Placebo	5	Some concerns	Low risk	No concerns	No concerns	Major concerns	Major concerns	Low	["Within-study bias","Heterogeneity","Incoherence"]
GLP-1:Placebo	3	Some concerns	Low risk	No concerns	Major concerns	No concerns	Major concerns	Low	["Within-study bias","Imprecision","Incoherence"]
Placebo:Resmetirom	2	No concerns	Low risk	No concerns	Major concerns	No concerns	Major concerns	Low	["Imprecision","Incoherence"]
FGF21:GLP-1	0	Some concerns	Low risk	No concerns	Major concerns	No concerns	Major concerns	Low	["Within-study bias","Imprecision","Incoherence"]
FGF21:Resmetirom	0	Some concerns	Low risk	No concerns	Major concerns	No concerns	Major concerns	Low	["Within-study bias","Imprecision","Incoherence"]
GLP-1:Resmetirom	0	No concerns	Low risk	No concerns	Major concerns	No concerns	Major concerns	Low	["Imprecision","Incoherence"]

Table S8.3 Hepatic fat reduction (MRI PDFF)

Comparison	Number of studies	Within-study bias	Reporting bias	Indirectness	Imprecision	Heterogeneity	Incoherence	Confidence rating	Reason(s) for downgrading
FGF21:Placebo	4	No concerns	Low risk	No concerns	No concerns	Major concerns	Major concerns	Low	["Heterogeneity","Incoherence"]
GLP-1:Placebo	4	No concerns	Low risk	No concerns	No concerns	Major concerns	Major concerns	Low	["Heterogeneity","Incoherence"]
Placebo:Resmetirom	3	No concerns	Low risk	No concerns	No concerns	No concerns	Major concerns	Low	["Incoherence"]
FGF21:GLP-1	0	No concerns	Low risk	No concerns	Major concerns	No concerns	Major concerns	Low	["Imprecision","Incoherence"]
FGF21:Resmetirom	0	No concerns	Low risk	No concerns	No concerns	Major concerns	Major concerns	Low	["Heterogeneity","Incoherence"]
GLP-1:Resmetirom	0	No concerns	Low risk	No concerns	No concerns	No concerns	Major concerns	Low	["Incoherence"]

Table S8.4 >30% Fat Reduction on MRI-PDFF

Comparison	Number of studies	Within-study bias	Reporting bias	Indirectness	Imprecision	Heterogeneity	Incoherence	Confidence rating	Reason(s) for downgrading
FGF21:Placebo	6	Some concerns	Low risk	No concerns	No concerns	No concerns	Major concerns	Low	["Within-study bias","Incoherence"]
GLP-1:Placebo	4	Some concerns	Low risk	No concerns	No concerns	Major concerns	Major concerns	Low	["Within-study bias","Heterogeneity","Incoherence"]
Placebo:Resmetirom	2	No concerns	Low risk	No concerns	No concerns	No concerns	Major concerns	Low	["Incoherence"]
FGF21:GLP-1	0	Some concerns	Low risk	No concerns	Major concerns	No concerns	Major concerns	Low	["Within-study bias","Imprecision","Incoherence"]
FGF21:Resmetirom	0	No concerns	Low risk	No concerns	Major concerns	No concerns	Major concerns	Low	["Imprecision","Incoherence"]
GLP-1:Resmetirom	0	No concerns	Low risk	No concerns	No concerns	Major concerns	Major concerns	Low	["Heterogeneity","Incoherence"]

Table S8.5 Change in VCTE

Comparison	Number of studies	Within-study bias	Reporting bias	Indirectness	Imprecision	Heterogeneity	Incoherence	Confidence rating	Reason(s) for downgrading
FGF21:Placebo	4	No concerns	Low risk	No concerns	No concerns	Major concerns	Major concerns	Low	["Heterogeneity","Incoherence"]
GLP-1:Placebo	4	No concerns	Low risk	No concerns	Major concerns	No concerns	Major concerns	Low	["Imprecision","Incoherence"]
Placebo:Resmetirom	2	Some concerns	Low risk	No concerns	Major concerns	No concerns	Major concerns	Low	["Within-study bias","Imprecision","Incoherence"]
FGF21:GLP-1	0	No concerns	Low risk	No concerns	Major concerns	No concerns	Major concerns	Low	["Imprecision","Incoherence"]
FGF21:Resmetirom	0	No concerns	Low risk	No concerns	Major concerns	No concerns	Major concerns	Low	["Imprecision","Incoherence"]
GLP-1:Resmetirom	0	No concerns	Low risk	No concerns	Major concerns	No concerns	Major concerns	Low	["Imprecision","Incoherence"]

Table S8.6 Change in ALT

Comparison	Number of studies	Within-study bias	Reporting bias	Indirectness	Imprecision	Heterogeneity	Incoherence	Confidence rating	Reason(s) for downgrading
FGF21:Placebo	5	No concerns	Low risk	No concerns	No concerns	No concerns	Major concerns	Low	["Incoherence"]
GLP-1:Placebo	6	No concerns	Low risk	No concerns	No concerns	Major concerns	Major concerns	Low	["Heterogeneity","Incoherence"]
Placebo:Resmetirom	2	Some concerns	Low risk	No concerns	No concerns	No concerns	Major concerns	Low	["Within-study bias","Incoherence"]
FGF21:GLP-1	0	No concerns	Low risk	No concerns	Major concerns	No concerns	Major concerns	Low	["Imprecision","Incoherence"]
FGF21:Resmetirom	0	Some concerns	Low risk	No concerns	Major concerns	No concerns	Major concerns	Low	["Within-study bias","Imprecision","Incoherence"]
GLP-1:Resmetirom	0	Some concerns	Low risk	No concerns	Major concerns	No concerns	Major concerns	Low	["Within-study bias","Imprecision","Incoherence"]

Table S8.7 Change in AST

Comparison	Number of studies	Within-study bias	Reporting bias	Indirectness	Imprecision	Heterogeneity	Incoherence	Confidence rating	Reason(s) for downgrading
FGF21:Placebo	5	No concerns	Low risk	No concerns	No concerns	Major concerns	Major concerns	Low	["Heterogeneity","Incoherence"]
GLP-1:Placebo	6	No concerns	Low risk	No concerns	No concerns	Major concerns	Major concerns	Low	["Heterogeneity","Incoherence"]
Placebo:Resmetirom	2	Some concerns	Low risk	No concerns	No concerns	Major concerns	Major concerns	Low	["Within-study bias","Heterogeneity","Incoherence"]
FGF21:GLP-1	0	No concerns	Low risk	No concerns	Major concerns	No concerns	Major concerns	Low	["Imprecision","Incoherence"]
FGF21:Resmetirom	0	Some concerns	Low risk	No concerns	Major concerns	No concerns	Major concerns	Low	["Within-study bias","Imprecision","Incoherence"]
GLP-1:Resmetirom	0	Some concerns	Low risk	No concerns	Major concerns	No concerns	Major concerns	Low	["Within-study bias","Imprecision","Incoherence"]

Table S8.8 Change in GGT

Comparison	Number of studies	Within-study bias	Reporting bias	Indirectness	Imprecision	Heterogeneity	Incoherence	Confidence rating	Reason(s) for downgrading
FGF21:Placebo	2	No concerns	Low risk	No concerns	Major concerns	No concerns	Major concerns	Low	["Imprecision","Incoherence"]
GLP-1:Placebo	4	No concerns	Low risk	No concerns	No concerns	Major concerns	Major concerns	Low	["Heterogeneity","Incoherence"]
Placebo:Resmetirom	2	Some concerns	Low risk	No concerns	No concerns	Major concerns	Major concerns	Low	["Within-study bias","Heterogeneity","Incoherence"]
FGF21:GLP-1	0	No concerns	Low risk	No concerns	Major concerns	No concerns	Major concerns	Low	["Imprecision","Incoherence"]
FGF21:Resmetirom	0	No concerns	Low risk	No concerns	Major concerns	No concerns	Major concerns	Low	["Imprecision","Incoherence"]
GLP-1:Resmetirom	0	No concerns	Low risk	No concerns	Major concerns	No concerns	Major concerns	Low	["Imprecision","Incoherence"]

Table S8.9 Adverse events

Comparison	Number of studies	Within-study bias	Reporting bias	Indirectness	Imprecision	Heterogeneity	Incoherence	Confidence rating	Reason(s) for downgrading
FGF21:Placebo	6	No concerns	Low risk	No concerns	Major concerns	No concerns	Major concerns	Low	["Imprecision","Incoherence"]
GLP-1:Placebo	5	Some concerns	Low risk	No concerns	Major concerns	No concerns	Major concerns	Low	["Within-study bias","Imprecision","Incoherence"]
Placebo:Resmetirom	2	No concerns	Low risk	No concerns	No concerns	No concerns	Major concerns	Low	["Incoherence"]
FGF21:GLP-1	0	No concerns	Low risk	No concerns	Major concerns	No concerns	Major concerns	Low	["Imprecision","Incoherence"]
FGF21:Resmetirom	0	No concerns	Low risk	No concerns	Major concerns	No concerns	Major concerns	Low	["Imprecision","Incoherence"]
GLP-1:Resmetirom	0	No concerns	Low risk	No concerns	Major concerns	No concerns	Major concerns	Low	["Imprecision","Incoherence"]

Table S8.10 Treatment discontinuation

Comparison	Number of studies	Within-study bias	Reporting bias	Indirectness	Imprecision	Heterogeneity	Incoherence	Confidence rating	Reason(s) for downgrading
FGF21:Placebo	6	No concerns	Low risk	No concerns	Major concerns	No concerns	Major concerns	Low	["Imprecision","Incoherence"]
GLP-1:Placebo	5	Some concerns	Low risk	No concerns	Major concerns	No concerns	Major concerns	Low	["Within-study bias","Imprecision","Incoherence"]
Placebo:Resmetirom	2	No concerns	Low risk	No concerns	No concerns	No concerns	Major concerns	Low	["Incoherence"]
FGF21:GLP-1	0	No concerns	Low risk	No concerns	Major concerns	No concerns	Major concerns	Low	["Imprecision","Incoherence"]
FGF21:Resmetirom	0	No concerns	Low risk	No concerns	Major concerns	No concerns	Major concerns	Low	["Imprecision","Incoherence"]
GLP-1:Resmetirom	0	No concerns	Low risk	No concerns	Major concerns	No concerns	Major concerns	Low	["Imprecision","Incoherence"]

Table S8.11 Nausea

Comparison	Number of studies	Within-study bias	Reporting bias	Indirectness	Imprecision	Heterogeneity	Incoherence	Confidence rating	Reason(s) for downgrading
FGF21:Placebo	6	No concerns	Low risk	No concerns	Major concerns	No concerns	Major concerns	Low	["Imprecision","Incoherence"]
GLP-1:Placebo	4	No concerns	Low risk	No concerns	No concerns	Major concerns	Major concerns	Low	["Heterogeneity","Incoherence"]
Placebo:Resmetirom	2	No concerns	Low risk	No concerns	No concerns	Major concerns	Major concerns	Low	["Heterogeneity","Incoherence"]
FGF21:GLP-1	0	No concerns	Low risk	No concerns	Major concerns	No concerns	Major concerns	Low	["Imprecision","Incoherence"]
FGF21:Resmetirom	0	No concerns	Low risk	No concerns	Major concerns	No concerns	Major concerns	Low	["Imprecision","Incoherence"]
GLP-1:Resmetirom	0	No concerns	Low risk	No concerns	Major concerns	No concerns	Major concerns	Low	["Imprecision","Incoherence"]

Table S8.12 Diarrhea

Comparison	Number of studies	Within-study bias	Reporting bias	Indirectness	Imprecision	Heterogeneity	Incoherence	Confidence rating	Reason(s) for downgrading
FGF21:Placebo	6	No concerns	Low risk	No concerns	No concerns	No concerns	Major concerns	Low	["Incoherence"]
GLP-1:Placebo	5	Some concerns	Low risk	No concerns	No concerns	Major concerns	Major concerns	Low	["Within-study bias","Heterogeneity","Incoherence"]
Placebo:Resmetirom	2	No concerns	Low risk	No concerns	No concerns	No concerns	Major concerns	Low	["Incoherence"]
FGF21:GLP-1	0	No concerns	Low risk	No concerns	Major concerns	No concerns	Major concerns	Low	["Imprecision","Incoherence"]
FGF21:Resmetirom	0	No concerns	Low risk	No concerns	Major concerns	No concerns	Major concerns	Low	["Imprecision","Incoherence"]
GLP-1:Resmetirom	0	No concerns	Low risk	No concerns	Major concerns	No concerns	Major concerns	Low	["Imprecision","Incoherence"]

Supplement S9: Summary of the excluded studies:

Table S9: Summary of the excluded studies

Study	Reason
Harrison,2020[1]	Not the intervention of interest
Ratziu,2016[2]	Not the intervention of interest
Khoo,2019[3]	Not the study design of interest
Metzner,2022[4]	Not the intervention of interest
Javanbakht,2023[5]	Not the study design of interest
Brown,2023[6]	Not the study design of interest
Lu,2024[7]	Not the study design of interest
Zhao,2023[8]	Not the study design of interest

Supplement S10: Meta-regression:

Figure S10.1 NASH resolution

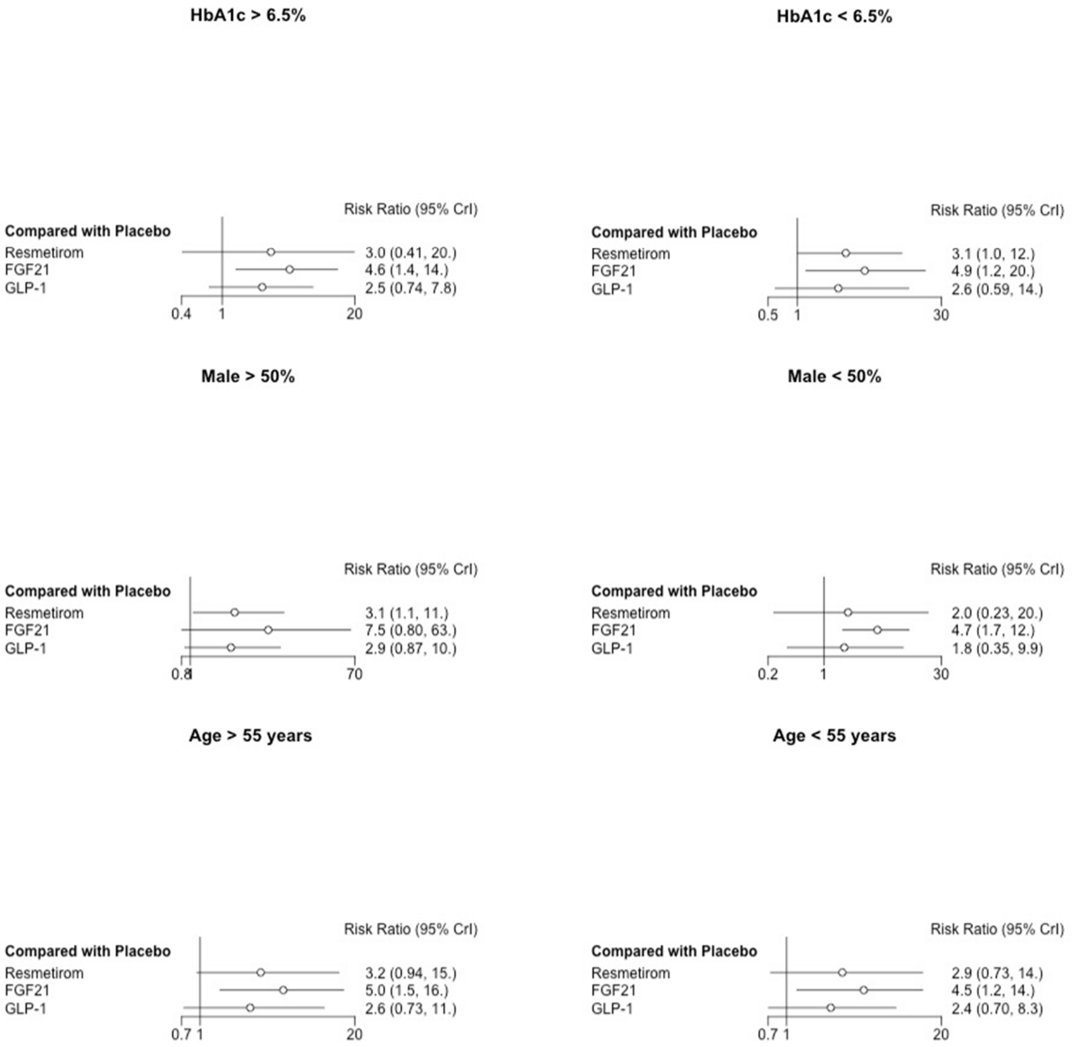

Figure S10.2 Improvement in Fibrosis

HbA1c > 6.5%

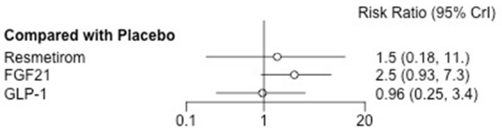

HbA1c < 6.5%

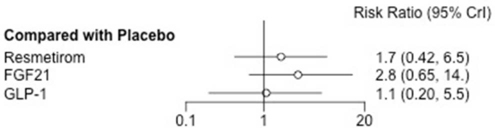

Male > 50%

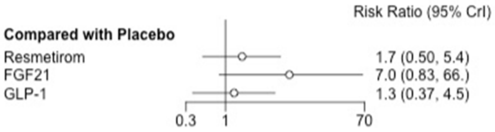

Male < 50%

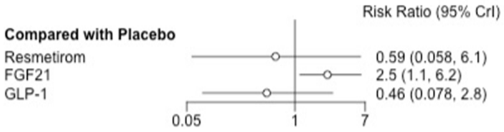

Age > 55 years

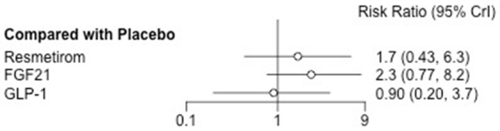

Age < 55 years

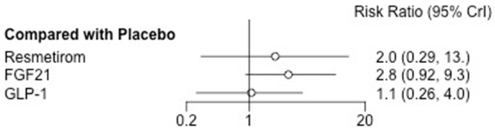

Figure S10.3 Hepatic fat reduction (MRI PDFF)

HbA1c < 6.5%

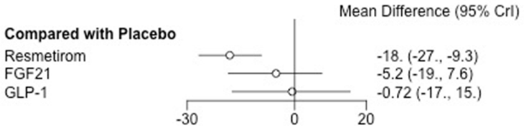

HbA1c > 6.5%

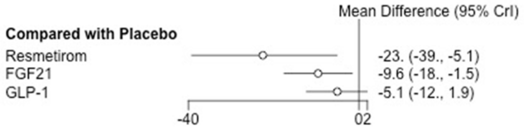

Male > 50%

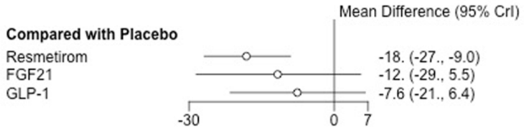

Male < 50%

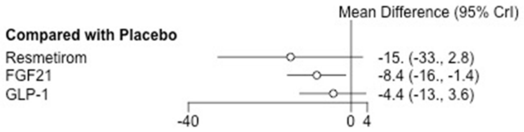

Age > 55 years

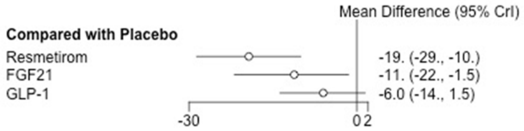

Age < 55 years

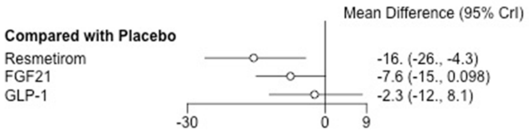

Figure S10.4 >30% Fat Reduction on MRI-PDFF

HbA1c > 6.5%

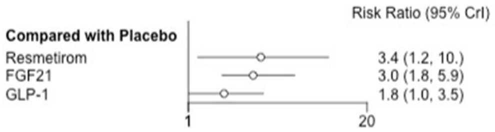

HbA1c < 6.5%

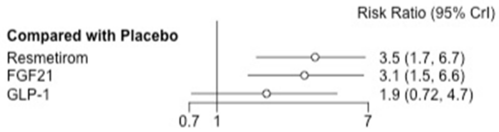

Male < 50%

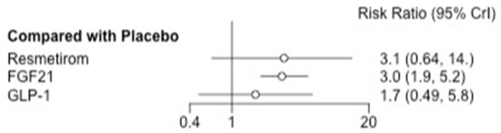

Male > 50%

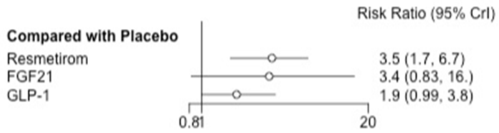

Age < 55 years

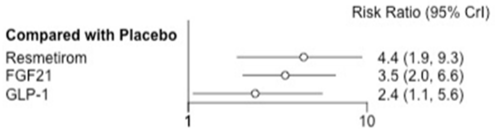

Age > 55 years

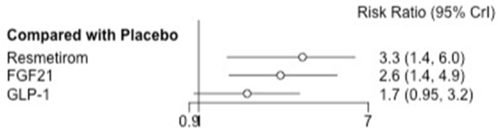

Figure S10.5 Change in VCTE

HbA1c > 6.5%

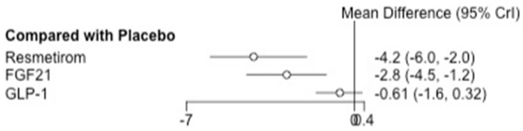

HbA1c < 6.5%

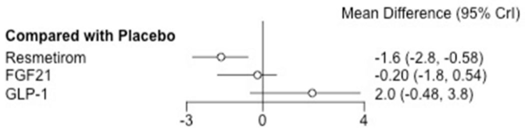

Male > 50%

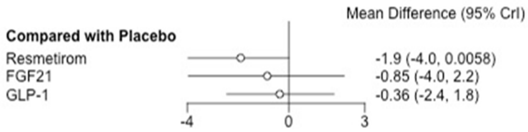

Male < 50%

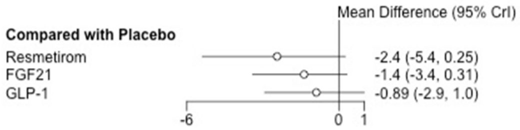

Age > 55 years

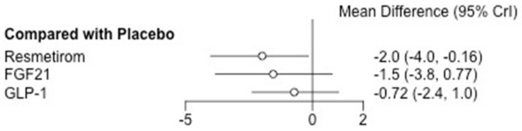

Age < 55 years

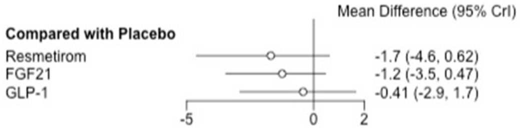

Figure S10.6 Change in ALT

HbA1c > 6.5%

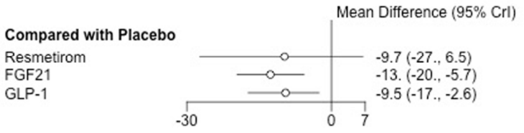

HbA1c < 6.5%

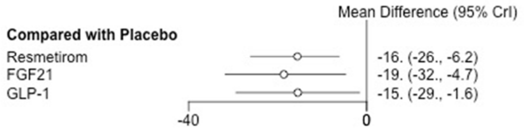

Male > 50%

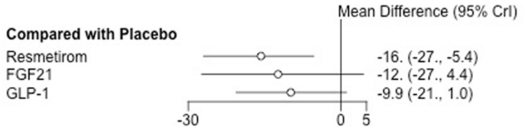

Male < 50%

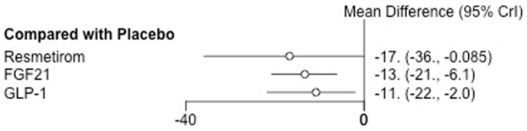

Age > 55 years

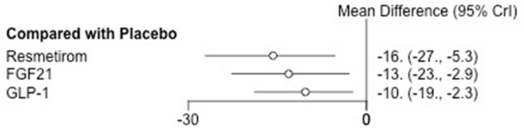

Age < 55 years

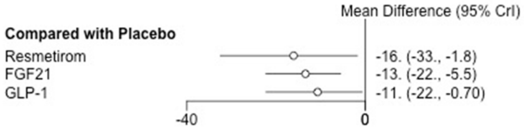

Figure S10.7 Change in AST

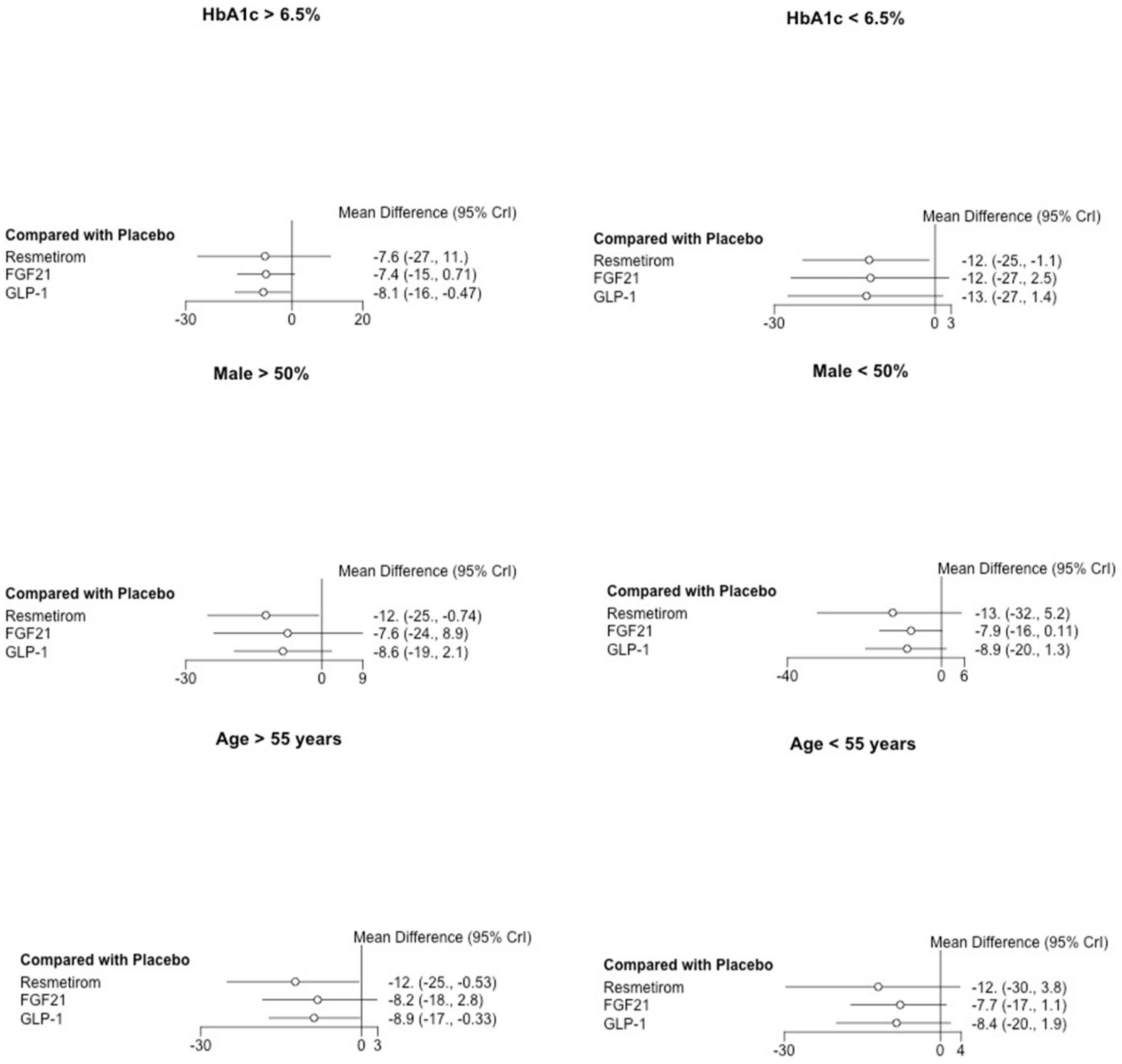

Figure S10.8 Change in GGT

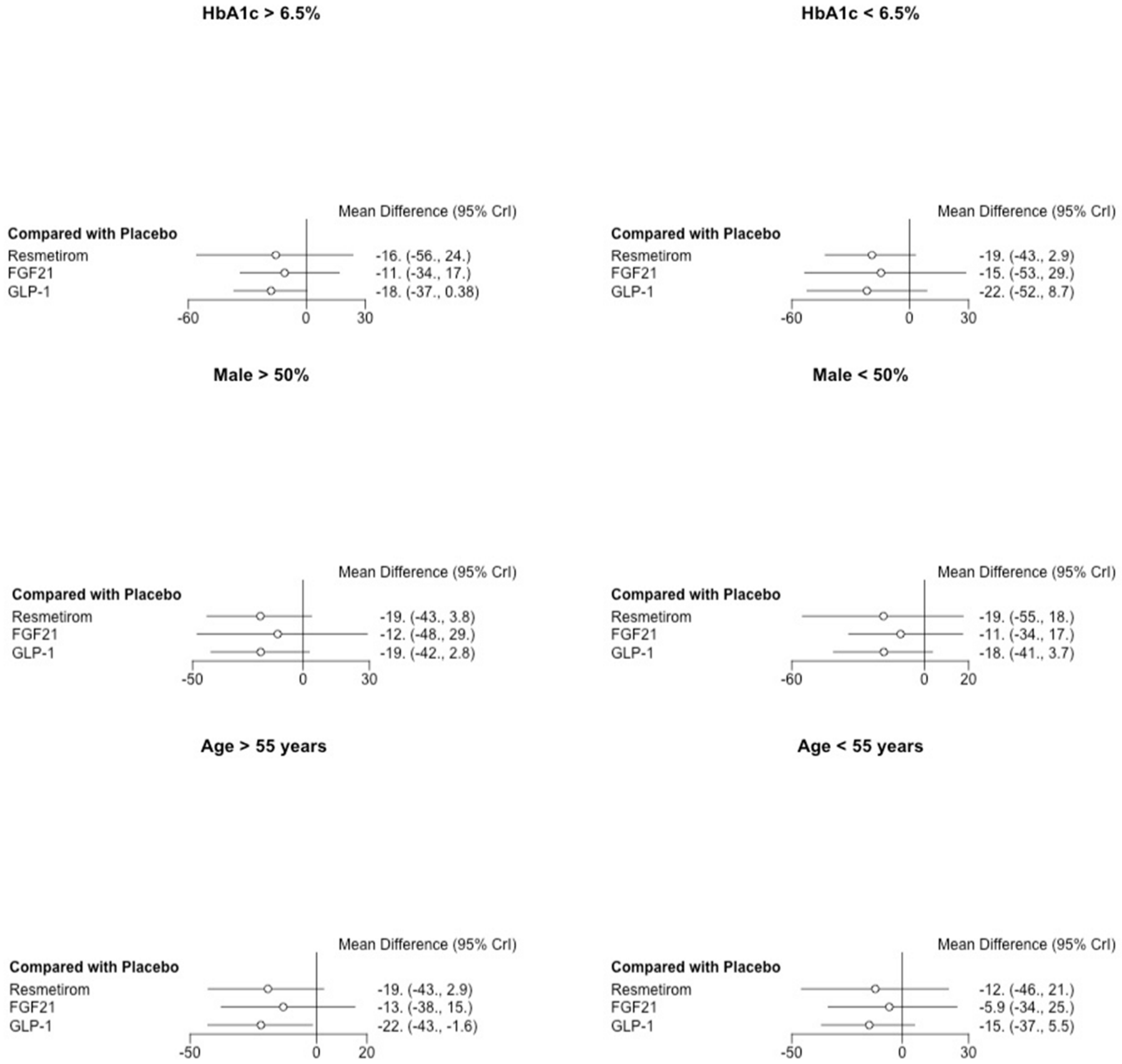

Figure S10.9 Adverse events

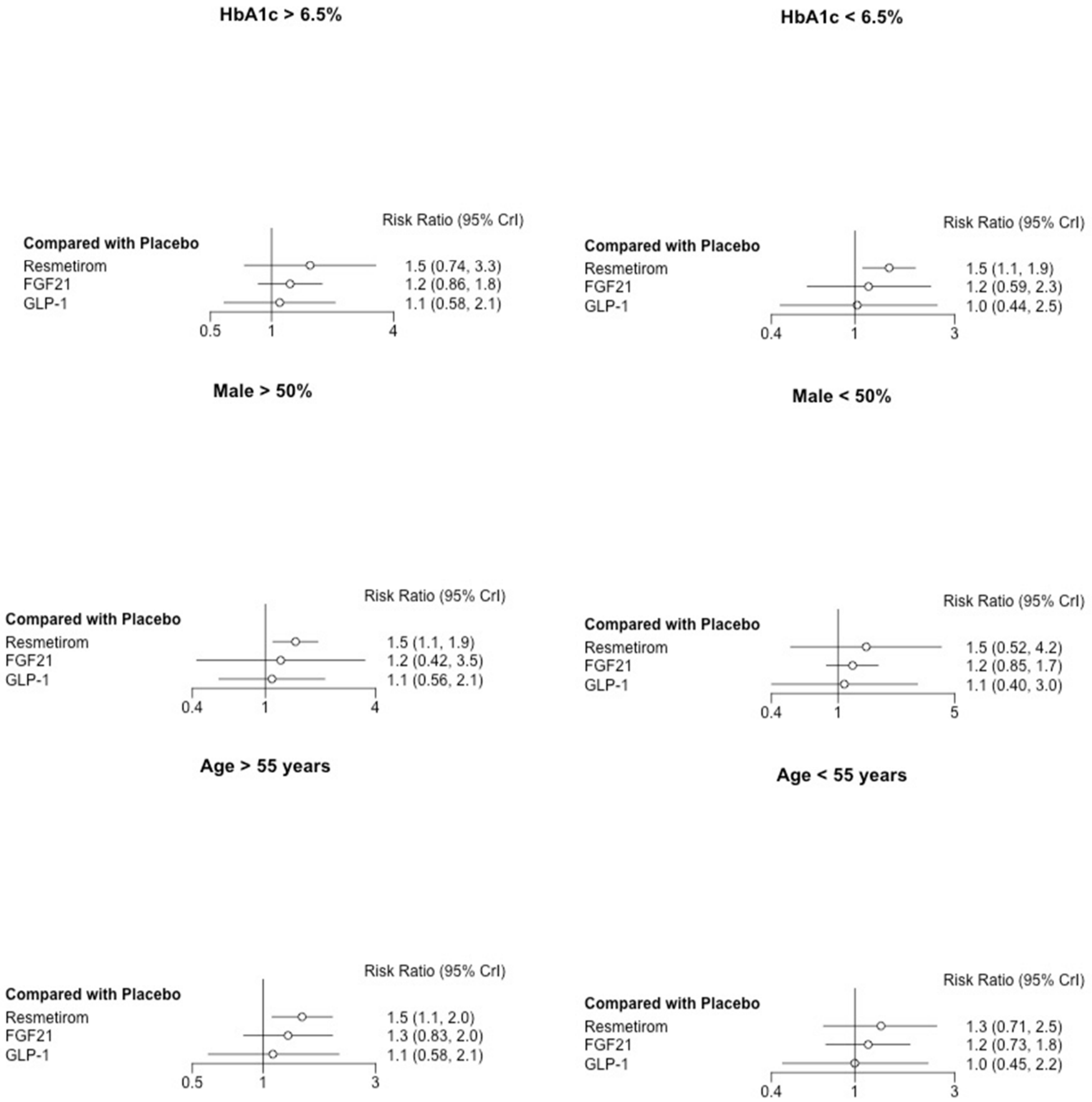

Figure S10.10 Treatment discontinuation

HbA1c > 6.5%

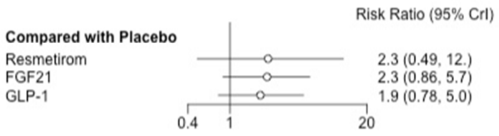

HbA1c < 6.5%

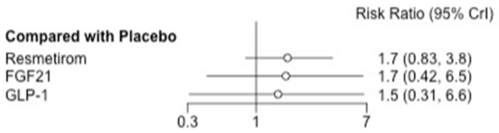

Male > 50%

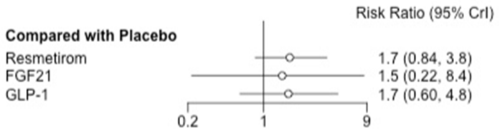

Male < 50%

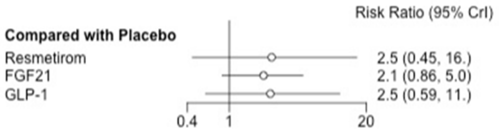

Age > 55 years

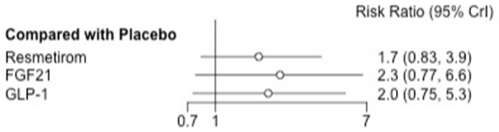

Age < 55 years

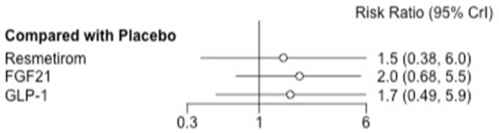

Figure S10.11 Nausea

HbA1c > 6.5%

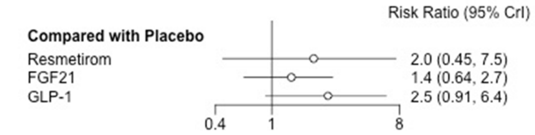

HbA1c < 6.5%

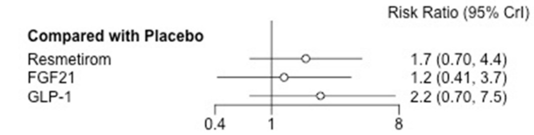

Male > 50%

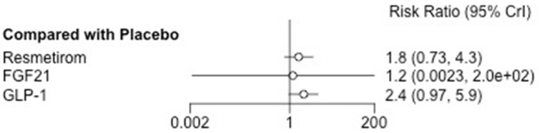

Male < 50%

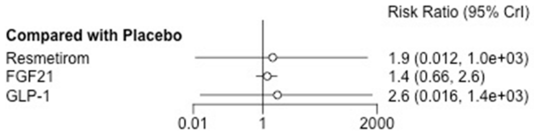

Age > 55 years

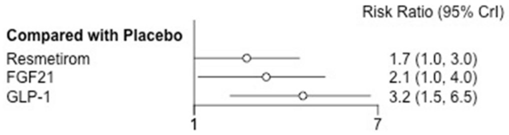

Age < 55 years

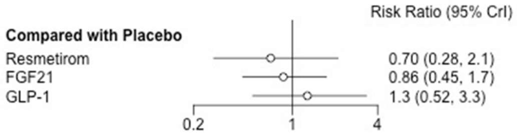

Figure S10.12 Diarrhea

HbA1c > 6.5%

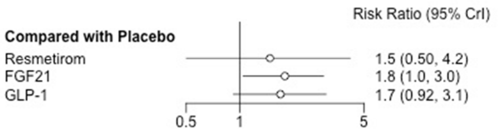

HbA1c < 6.5%

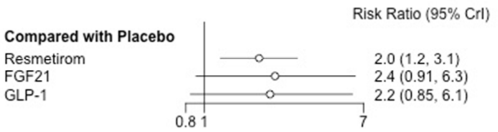

Male > 50%

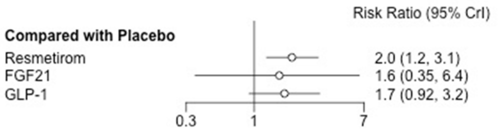

Male < 50%

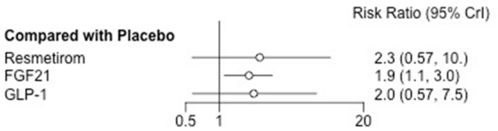

Age > 55 years

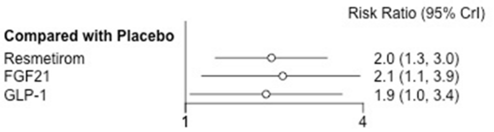

Age < 55 years

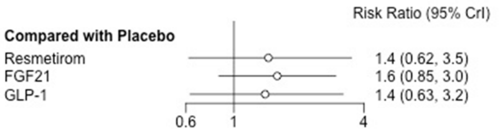

Supplement S11. PRISMA checklist

Section and Topic	Item #	Checklist item	Location where item is reported
**TITLE**			
Title	1	Identify the report as a systematic review.	1
**ABSTRACT**			
Abstract	2	See the PRISMA 2020 for Abstracts checklist.	1
**INTRODUCTION**			
Rationale	3	Describe the rationale for the review in the context of existing knowledge.	2
Objectives	4	Provide an explicit statement of the objective(s) or question(s) the review addresses.	2
**METHODS**			
Eligibility criteria	5	Specify the inclusion and exclusion criteria for the review and how studies were grouped for the syntheses.	3,4
Information sources	6	Specify all databases, registers, websites, organisations, reference lists and other sources searched or consulted to identify studies. Specify the date when each source was last searched or consulted.	3,4
Search strategy	7	Present the full search strategies for all databases, registers and websites, including any filters and limits used.	3,4
Selection process	8	Specify the methods used to decide whether a study met the inclusion criteria of the review, including how many reviewers screened each record and each report retrieved, whether they worked independently, and if applicable, details of automation tools used in the process.	3,4
Data collection process	9	Specify the methods used to collect data from reports, including how many reviewers collected data from each report, whether they worked independently, any processes for obtaining or confirming data from study investigators, and if applicable, details of automation tools used in the process.	3,4
Data items	10a	List and define all outcomes for which data were sought. Specify whether all results that were compatible with each outcome domain in each study were sought (e.g. for all measures, time points, analyses), and if not, the methods used to decide which results to collect.	3,4
	10b	List and define all other variables for which data were sought (e.g. participant and intervention characteristics, funding sources). Describe any assumptions made about any missing or unclear	3,4

Supplement S11. PRISMA checklist

Section and Topic	Item #	Checklist item	Location where item is reported
		information.	
Study risk of bias assessment	11	Specify the methods used to assess risk of bias in the included studies, including details of the tool(s) used, how many reviewers assessed each study and whether they worked independently, and if applicable, details of automation tools used in the process.	3,4
Effect measures	12	Specify for each outcome the effect measure(s) (e.g. risk ratio, mean difference) used in the synthesis or presentation of results.	3,4
Synthesis methods	13a	Describe the processes used to decide which studies were eligible for each synthesis (e.g. tabulating the study intervention characteristics and comparing against the planned groups for each synthesis.	4
	13b	Describe any methods required to prepare the data for presentation or synthesis, such as handling of missing summary statistics, or data conversions.	4
	13c	Describe any methods used to tabulate or visually display results of individual studies and syntheses.	4
	13d	Describe any methods used to synthesize results and provide a rationale for the choice(s). If meta-analysis was performed, describe the model(s), method(s) to identify the presence and extent of statistical heterogeneity, and software package(s) used.	4
	13e	Describe any methods used to explore possible causes of heterogeneity among study results (e.g. subgroup analysis, meta-regression).	4
	13f	Describe any sensitivity analyses conducted to assess robustness of the synthesized results.	4
Reporting bias assessment	14	Describe any methods used to assess risk of bias due to missing results in a synthesis (arising from reporting biases).	4
Certainty assessment	15	Describe any methods used to assess certainty (or confidence) in the body of evidence for an outcome.	4
**RESULTS**			
Study selection	16a	Describe the results of the search and selection process, from the number of records identified in the search to the number of studies included in the review, ideally using a flow diagram.	4,supplement, figure 1
	16b	Cite studies that might appear to meet the inclusion criteria, but which were excluded, and explain why they were excluded.	4

Supplement S11. PRISMA checklist

Section and Topic	Item #	Checklist item	Location where item is reported
Study characteristics	17	Cite each included study and present its characteristics.	4,supplement
Risk of bias in studies	18	Present assessments of risk of bias for each included study.	4,supplement
Results of individual studies	19	For all outcomes, present, for each study: (a) summary statistics for each group (where appropriate) and (b) an effect estimate and its precision (e.g. confidence/credible interval), ideally using structured tables or plots.	4-6
Results of syntheses	20a	For each synthesis, briefly summarise the characteristics and risk of bias among contributing studies.	4-6
	20b	Present results of all statistical syntheses conducted. If meta-analysis was done, present for each the summary estimate and its precision (e.g. confidence/credible interval) and measures of statistical heterogeneity. If comparing groups, describe the direction of the effect.	4-6
	20c	Present results of all investigations of possible causes of heterogeneity among study results.	4-6
	20d	Present results of all sensitivity analyses conducted to assess the robustness of the synthesized results.	4, supplement
Reporting biases	21	Present assessments of risk of bias due to missing results (arising from reporting biases) for each synthesis assessed.	4, supplement
Certainty of evidence	22	Present assessments of certainty (or confidence) in the body of evidence for each outcome assessed.	4,supplement
**DISCUSSION**			
Discussion	23a	Provide a general interpretation of the results in the context of other evidence.	8,9
	23b	Discuss any limitations of the evidence included in the review.	8,9
	23c	Discuss any limitations of the review processes used.	8,9
	23d	Discuss implications of the results for practice, policy, and future research.	8,9
**OTHER INFORMATION**			
Registration and protocol	24a	Provide registration information for the review, including register name and registration number, or state that the review was not registered.	3

Supplement S11. PRISMA checklist

**Section and Topic**	**Item #**	**Checklist item**	**Location where item is reported**
	24b	Indicate where the review protocol can be accessed, or state that a protocol was not prepared.	3
	24c	Describe and explain any amendments to information provided at registration or in the protocol.	3
Support	25	Describe sources of financial or non-financial support for the review, and the role of the funders or sponsors in the review.	9
Competing interests	26	Declare any competing interests of review authors.	9
Availability of data, code and other materials	27	Report which of the following are publicly available and where they can be found: template data collection forms; data extracted from included studies; data used for all analyses; analytic code; any other materials used in the review.	9

References

1. Harrison, S.A.; Alkhouri, N.; Davison, B.A.; Sanyal, A.; Edwards, C.; Colca, J.R.; Lee, B.H.; Loomba, R.; Cusi, K.; Kolterman, O.; et al. Insulin sensitizer MSDC-0602K in non-alcoholic steatohepatitis: A randomized, double-blind, placebo-controlled phase IIb study. *J. Hepatol.* **2020**, *72*, 613–626. https://doi.org/10.1016/j.jhep.2019.10.023.
2. Ratziu, V.; Harrison, S.A.; Francque, S.; Bedossa, P.; Lehert, P.; Serfaty, L.; Romero-Gomez, M.; Boursier, J.; Abdelmalek, M.; Caldwell, S.; et al. Elafibranor, an Agonist of the Peroxisome Proliferator-Activated Receptor-α and -δ, Induces Resolution of Nonalcoholic Steatohepatitis Without Fibrosis Worsening. *Gastroenterology* **2016**, *150*, 1147–1159.e5. https://doi.org/10.1053/j.gastro.2016.01.038.
3. Khoo, J.; Hsiang, J.C.; Taneja, R.; Koo, S.H.; Soon, G.H.; Kam, C.J.; Law, N.M.; Ang, T.L. Randomized trial comparing effects of weight loss by liraglutide with lifestyle modification in non-alcoholic fatty liver disease. *Liver Int.* **2019**, *39*, 941–949. https://doi.org/10.1111/liv.14065.
4. Metzner, V.; Herzog, G.; Heckel, T.; Bischler, T.; Hasinger, J.; Otto, C.; Fassnacht, M.; Geier, A.; Seyfried, F.; Dischinger, U. Liraglutide + PYY(3-36) Combination Therapy Mimics Effects of Roux-en-Y Bypass on Early NAFLD Whilst Lacking-Behind in Metabolic Improvements. *J. Clin. Med.* **2022**, *11*. https://doi.org/10.3390/jcm11030753.
5. Javanbakht, M.; Fishman, J.; Moloney, E.; Rydqvist, P.; Ansaripour, A. Early Cost-Effectiveness and Price Threshold Analyses of Resmetirom: An Investigational Treatment for Management of Nonalcoholic Steatohepatitis. *Pharmacoecon Open* **2023**, *7*, 93–110. https://doi.org/10.1007/s41669-022-00370-2.
6. Brown, E.A.; Minnich, A.; Sanyal, A.J.; Loomba, R.; Du, S.; Schwarz, J.; Ehman, R.L.; Karsdal, M.; Leeming, D.J.; Cizza, G.; et al. Effect of pegbelfermin on NASH and fibrosis-related biomarkers and correlation with histological response in the FALCON 1 trial. *JHEP Rep.* **2023**, *5*, 100661. https://doi.org/10.1016/j.jhepr.2022.100661.
7. Lu, Y.; Yu, B.; Bu, Y.; Lou, J.; Jin, Y. Pegbelfermin for reducing transaminase levels in patients with non-alcoholic steatohepatitis: A dose-response meta-analysis of randomized controlled trials. *Front. Med.* **2024**, *11*, 1293336. https://doi.org/10.3389/fmed.2024.1293336.
8. Zhao, Y.; Zhao, W.; Bu, H.; Toshiyoshi, M.; Zhao, Y. Liraglutide on type 2 diabetes mellitus with nonalcoholic fatty liver disease: A systematic review and meta-analysis of 16 RCTs. *Medicine* **2023**, *102*, e32892. https://doi.org/10.1097/md.0000000000032892.
